# Multi‐Omics Analysis Reveals Photodynamic Therapy Ameliorating Skin Photoaging by Improving Cellular Senescence Through Mitohormesis‐Mediated Reduction of Citrate Content

**DOI:** 10.1111/acel.70328

**Published:** 2025-12-28

**Authors:** Yu Yan, Qihang Chang, Yun Wu, Yiting Zhao, Guorong Yan, Zhi Cao, Haiyan Zhang, Xiuli Wang, Qingyu Zeng, Peiru Wang

**Affiliations:** ^1^ Institute of Photomedicine, Shanghai Skin Disease Hospital Tongji University School of Medicine Shanghai China

**Keywords:** ALA‐PDT, cellular senescence, mitohormesis, photoaging, reactive oxygen species

## Abstract

Clinical evidence supports the anti‐photoaging efficacy of 5‐aminolevulinic acid photodynamic therapy (ALA‐PDT), yet its mechanism remains elusive. Paradoxically, ALA‐PDT generates reactive oxygen species (ROS), a key mediator of ultraviolet radiation (UVR)‐induced photoaging, raising questions about its rejuvenating effects. Here, we employed a multi‐omics approach to clarify this paradox. A UVR‐induced hairless mouse model of photoaging was treated with ALA‐PDT, followed by transcriptomic, proteomic, and metabolomic profiling of skin biopsies. In vitro, fibroblast senescence was induced by UV irradiation to evaluate ALA‐PDT's protective effects. Mitochondrial function and citrate (CA) levels were assessed pre‐ and post‐treatment. ALA‐PDT significantly ameliorated photoaging phenotypes in mice, with multi‐omics data revealing sustained improvements in epidermal structure, extracellular matrix integrity, and immune responses. Key mechanistic findings included ALA‐PDT‐induced mitohormesis and tricarboxylic acid cycle reprogramming, notably reduced intracellular CA. In vitro, low‐dose ALA‐PDT downregulated senescence markers and CA content in UV‐stressed fibroblasts, concomitant with upregulated mitohormesis markers. These effects were abrogated by inhibiting mitochondrial ROS, suggesting ROS‐dependent mitohormetic signaling. Collectively, our data demonstrate that low‐dose ALA‐PDT alleviates photoaging by mitigating cellular senescence via mitohormesis‐mediated CA reduction, offering a novel metabolic intervention strategy for age‐related skin disorders.

## Graphical abstract

Ultraviolet‐induced skin photoaging involves cellular senescence. Low‐dose photodynamic therapy (PDT) counteracts this by triggering mitochondrial ROS and mitohormesis, which improve metabolism and ameliorate senescence, leading to reduced wrinkles and enhanced skin barrier.

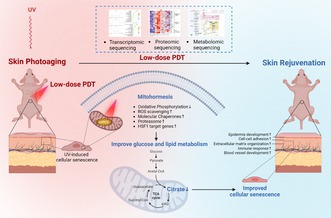

## 1 Introduction

The skin, as the largest and outermost organ, undergoes aging influenced by intrinsic factors like genetics and extrinsic factors, with ultraviolet radiation (UVR) being a primary cause of extrinsic aging, commonly known as photoaging (Oppel and Korting 2004). UVR accelerates this process by generating reactive oxygen species (ROS), which damage DNA, proteins, and lipids, leading to wrinkles, pigmentation changes, and loss of elasticity. Photoaging has impacts on not only skin health but also overall health (Franco et al. 2022), mortality risk, and longevity (Gunn et al. 2016; Waaijer et al. 2012). Enhancing skin resistance to photoaging is thus vital for promoting both skin and systemic health.

5‐aminolevulinic acid photodynamic therapy (ALA‐PDT) is a new noninvasive combination treatment and diagnostic modality. From the end of the last century to the beginning of this century, topical PDT was first recognized as an effective treatment for actinic keratosis (AK) and squamous cell carcinoma (SCC), mainly caused by photoaging (Morton et al. 2002). Subsequently, it has been found that PDT may also have a therapeutic effect on photoaging (Morton et al. 2008). Consistently, our clinical study found that ALA‐PDT can improve localized photoaging in the long term while treating AK (Zhang et al. 2021). At present, the skin rejuvenation effect of PDT on photoaging skin has been widely recognized (Kohl et al. 2017; Monfrecola et al. 2021).

ALA is converted into protoporphyrin IX (PpIX), which is a photosensitizer capable of generating ROS upon light activation to exert its photosensitive effects (Shi et al. 2021). However, it seems to be contradictory that PDT exerts an antiaging effect by inducing ROS and free radicals in cells (Fonda‐Pascual et al. 2016; Jang et al. 2013), while UVR likewise exerts a pro‐aging effect also by causing oxidative stress in cells (Ansary et al. 2021).

Mitohormesis may explain the above contradiction. Mitohormesis refers to irritation of low levels of free radicals in mitochondria that increase cellular defenses through an adaptive response (Yun and Finkel 2014; Ristow and Zarse 2010). Many studies have shown that mitohormesis, which is triggered by the production of mitochondrial ROS, is the reason for prolonging the lifespan of mice, flies, and *Caenorhabditis elegans* (Ristow and Schmeisser 2011; Obata et al. 2018; Weimer et al. 2014; Zarse et al. 2012). Mitochondrial stress can cause the integrative stress response (ISR), and the common denominator of ISR is phosphorylation of the translation initiation factor eIF2α, which reduces overall total protein synthesis while allowing preferential translation of certain factors (Cheng et al. 2023). Activation of ISR simultaneously causes an unfolded protein response (UPR) (Anderson and Haynes 2020), which coordinates the expression of genes required for protein synthesis, resulting in a decrease in overall protein synthesis (Münch and Harper 2016). Boos et al. further found that mitochondrial “clogger” proteins increase gene expression encoding chaperones and components of the ubiquitin‐proteasome system by triggering the activation of the transcription factor HSF1, while inhibiting the cytosolic ribosomes and mitochondrial enzymes of oxidative phosphorylation (OXPHOS) and the tricarboxylic acid cycle (TCA cycle) (Boos et al. 2019).

In this study, we performed multi‐omics sequencing analysis and in vitro experimental verification, and found that low‐dose ALA‐PDT is able to alleviate skin photoaging by enhancing cellular rejuvenation through mitohormesis, which leads to a decrease in citrate (CA) levels.

## 2 Method

### 2.1 Animals and Low Dose ALA‐PDT Treatment

Hairless SKH‐1 mice (female, 6–8 weeks old, immunocompetent) were obtained from Shanghai Public Health Clinical. As mentioned in a previous study (Zhou et al. 2021), photoaging models are constructed with UVR irradiation (Solar UV Simulator, SIGMA, Shanghai, China) five times a week for 8 weeks. This equipment emitted a mixture of UVB and UVA rays, with an output power of UVB 3.5 mJ/cm^2^ and UVA 0.6 mJ/cm^2^. The irradiation time was 1 min during the 1st and 2nd weeks, 1 min and 10 s during the 3rd and 4th weeks, 1 min and 20 s during the 5th and 6th weeks, and 1 min and 30 s during the 7th and 8th weeks. By the 8th week, the establishment of the SKH‐1 mouse skin photoaging model was completed. Subsequently, in the 9th, 10th, and 11th weeks, the mice were exposed to UV light twice a week, with each session lasting 1 min and 30 s to maintain the photoaged phenotype.

The skin samples from the photoaged mice (Pho_age) used for sequencing were collected in the 11th week, coinciding with the 24‐h time point after treatment in the ALA‐PDT group. The ALA‐PDT group received additional ALA‐PDT treatments during the 8th–9th, 9th–10th, and 10th–11th weeks, with samples collected at 24 h, 1 week, and 1 month after the last treatment (Figure S1A). ALA‐PDT treatment is as previously described (Yang et al. 2022). Briefly, 3% ALA cream (Shanghai Fudan‐Zhangjiang Biopharmaceutical Co. Ltd., Shanghai, China) was evenly applied to the back of SKH‐1 mice and placed in a dark room protected from light for 3 h. After removing excess ALA cream, the dorsal skin was irradiated with 633 ± 15 nm LED light (Wuhan Yage Optic and Electronic Technique Co. Ltd., Wuhan, China) at 100 mW/cm^2^ for a total dose of 6.0 J.

Skin wrinkles were scored according to previously established methods, using the following criteria: 0—Smooth skin; 1—Fine wrinkle; 2—A little bit shallow wrinkles; 3—Shallow wrinkles across the dorsal skin; 4—Deep and coarse wrinkles with laxity; 5—Increased wrinkle depth; 6—Severe wrinkles with skin damage occurs (He et al. 2024).

### 2.2 Multi‐Omics Analysis

RNA sequencing, proteomics analysis, and metabolomic analysis were carried out by Shanghai Personal Biotechnology Cp. Ltd. The RNA concentration, quality, and integrity were assessed using a Nano Drop spectrophotometer (Thermo Scientific). Protein samples were chromatographically separated and analyzed by mass spectrometry using the timsTOF Pro mass spectrometer. Metabolomic samples were separated using a Vanquish LC ultra‐performance liquid chromatography system (UHPLC) and then used with Q Exactive series mass spectrometry. Mass spectrometry analysis was performed by electrospray ionization (ESI) positive ion and negative ion modes, respectively. Single‐cell sequencing data were obtained from the Bioproject database under the accession number PRJNA977409 (Yan et al. 2023).

The *DESeq2* r‐package (V1.40.2) was employed to identify genes that exhibit significant expressionariations, with a threshold of *p* value less than 0.05 to define such differentially expressed genes (DEGs). Also, proteins with a threshold of *p* value less than 0.05 were defined as differentially expressed proteins (DEPs). To elucidate the roles of these DEGs, functional annotation was conducted using the *clusterProfiler* r‐package (V4.8.3), which includes Gene Ontology (GO), Kyoto Encyclopedia of Genes and Genomes (KEGG) pathway, and gene set enrichment analysis (GSEA). Additionally, the *ComplexHeatmap* r‐package (V2.16.0) was utilized to graphically represent the expression levels of these genes across various sample groups. The *GSVA* r‐package (V1.48.3) was used to score function‐specific gene sets, which were obtained from the MSigDB database (https://www.gsea‐msigdb.org/gsea/msigdb). Combined analysis of transcriptome and metabolome was performed by MetaboAnalyst (https://www.metaboanalyst.ca). Schematic diagram of changes in glucose metabolism‐related genes and metabolite content (Figure 5E) was created by the BioRender (https://www.biorender.com/).

### 2.3 Skin Barrier Function Tests and Staining of Skin Sections

The trans epidermal water loss (TEWL) and MoistureMeter tools (Delfin Technologies, Finland) were applied to evaluate the skin barrier integrity in SKH‐1 mice. The collected skin samples underwent dehydration, preparation for sectioning, and subsequent staining with hematoxylin and eosin (HE) as well as Masson trichrome methods. Anti‐p21 antibody (ab188224, Abcam), anti‐p16 antibody (23200S, CST), anti‐lamin B1 antibody (ab16048, Abcam), and anti‐ki67 antibody (ab16667, Abcam) were used to immunohistochemically stain mouse skin tissue sections.

### 2.4 Cell Culture, UVB Radiation, and ALA‐PDT Therapy

Normal Human Epidermal Keratinocytes (NHEK) were obtained from Shanghai Kuisai Biotechnology Co. Ltd. NHEK, the human immortalized epidermal keratinocyte cell line (HaCaT, Shanghai Zhongqiao Xinzhou Biotechnology, China), and human dermal fibroblasts (HDF, Shanghai Daixuan Biotechnology, China) were maintained at 37°C with 5% CO_2_ in a growth medium consisting of DMEM (Hyclone, USA) supplemented with 10% fetal bovine serum (Gibco, CA) and 1% penicillin/streptomycin (100 U/mL, Gibco, CA).

For UVB irradiation, the cell culture medium was removed and only a thin layer of PBS was added. The dose of UVB irradiation was 100 mJ/cm^2^ (SH4 ultraviolet phototherapy instrument, SIGMA, Shanghai, China), and the subsequent treatment step was conducted 24 h post‐irradiation.

For ALA‐PDT therapy, 1 mM of ALA was added to a DMEM solution and incubated for 1 h in the dark, followed by exposure to different doses of 633 ± 15 nm LED light (Wuhan Yage Optic and Electronic Technique Co. Ltd., Wuhan, China). MitoSOX and JC‐1 assays were performed immediately after ALA‐PDT treatment, and other experiments were detected at 24 h after treatment.

### 2.5 Senescence‐Associated β‐Galactosidase Staining, qPCR, and Western Blot Analysis

Senescence β‐galactosidase (SA‐β‐gal) staining was detected by the kit (Beyotime, China) according to the manufacturer's instructions. RNA was extracted from the cells with Trizol and analyzed by real‐time fluorescence quantitative PCR. The following primers were used (Table 1).

**TABLE 1:** The primer sequences for RT‐qPCR.

Genes	Species	Forward primers	Reverse primers
CDKN1A	Human	5′‐TGTCCGTCAGAACCCATGC‐3′	5′‐AAAGTCGAAGTTCCATCGCTC‐3′
CDKN2A	Human	5′‐GATCCAGGTGGGTAGAAGGTC‐3′	5′‐CCCCTGCAAACTTCGTCCT‐3′
TP53	Human	5′‐CAGCACATGACGGAGGTTGT‐3′	5′‐TCATCCAAATACTCCACACGC‐3′
IDH2	Human	5′‐CTGGACGAGACGGTTCTCCT‐3′	5′‐CACCACGATCACCACTCTTG‐3′
EIF2S1	Human	5′‐GAAGGCGTATCCGTTCTATCAAC‐3′	5′‐AGCAACATGACGAAGAATGCTAT‐3′
IL1β	Human	5′‐TTCGACACATGGGATAACGAGG‐3′	5′‐TTTTTGCTGTGAGTCCCGGAG‐3′
IL6	Human	5′‐CCTGAACCTTCCAAAGATGGC‐3′	5′‐TTCACCAGGCAAGTCTCCTCA‐3′
IL8	Human	5′‐ACTGAGAGTGATTGAGAGTGGAC‐3′	5′‐AACCCTCTGCACCCAGTTTTC‐3′
MMP1	Human	5′‐CTCTGGAGTAATGTCACACCTCT‐3′	5′‐TGTTGGTCCACCTTTCATCTTC‐3′
TNFα	Human	5′‐CCTCTCTCTAATCAGCCCTCTG‐3′	5′‐GAGGACCTGGGAGTAGATGAG‐3′
ACTB	Human	5′‐CATGTACGTTGCTATCCAGGC‐3′	5′‐CTCCTTAATGTCACGCACGAT‐3′
Cdk2	Mouse	5′‐CTCTCACGGGCATTCCTCTTC‐3′	5′‐CCCTCTGCATTGATAAGCAGG‐3′
Cd28	Mouse	5′‐GTTCTTGGCTCTCAACTTCTTCT‐3′	5′‐TGAGGCTGACCTCGTTGCTAT‐3′
Itgav	Mouse	5′‐TTGATTCAACAGGCAATCGAGA‐3′	5′‐AGCATACTCAACGGTCTTTGTG‐3′
Actb	Mouse	5′‐GTGACGTTGACATCCGTAAAGA‐3′	5′‐GCCGGACTCATCGTACTCC‐3′

*Note:* The antibodies used in western blot analysis were as following: anti‐IDH2 (#15932‐1‐AP), anti‐EIF2S1 (#82936‐1‐RR), anti‐phospho‐EIF2S1 (#28740‐1‐AP), anti‐GAPDH (#60004‐1‐IG), anti‐beta‐Tublin (#DF7967), and anti‐Collagen I (#ab270993).

### 2.6 Cell Function Assays

Flow cytometry detection of mitochondrial ROS was performed using MitoSOX Green (Invitrogen, M36005) in accordance with standard bioassay protocols. Mitochondrial ROS levels were measured using MitoSOX Red (Invitrogen, M36007) and Hoechst 33,342 (#C1026). Mitoquinone mesylate (MedChemExpress, HY‐100116A) was used to clear mitochondrial ROS. CA (#C2404, Sigma‐Aldrich) was dissolved in phosphate‐buffered saline (PBS) to obtain stock solutions. CA content was determined by a citrate content detection kit (Solarbio, bc2150). Mitochondrial membrane potential assay kit with JC‐1 (Solarbio, M8650) was used to measure the mitochondrial membrane potential of cells. CCK‐8 Detection: Conducted using the CCK‐8 Kit (CCK8 reagent‐based cell viability assay) (Weiao, WH1199). Live/Dead Staining: Performed with the (Beyotime, C2015M) Calcein/PI Cell Viability and Cytotoxicity Assay Kit, enabling simultaneous visualization of viable (Calcein‐positive) and nonviable (PI‐positive) cells. ELISA kit‐based detection of changes in cell supernatant levels of IL‐1β (Weiao, EH10269M), IL‐6 (Weiao, EH10293M), IL‐8 (Weiao, EH10296S), MMP‐1 (Weiao, EH10350S), MMP‐3 (Weiao, EH10356M), and TNF‐α (Weiao, EH10497M). Seahorse XF24 assay for OCR (Oxygen Consumption Rate) and ECAR (Extracellular Acidification Rate) detection was conducted by Junli Biotechnology (Shanghai) Co. Ltd.

### 2.7 Statistical Analysis

The values that conform to a normal distribution were expressed as means ± standard deviation (SD). Unpaired *t*‐tests were employed to compare the two groups that conform to a normal distribution. GraphPad Prism 8 statistical software was used to compare the study groups. *p* Values less than 0.05 were considered statistically significant.

## 3 Results

### 3.1 ALA‐PDT Can Significantly Improve Skin Photoaging in Mice

**FIGURE 1:**
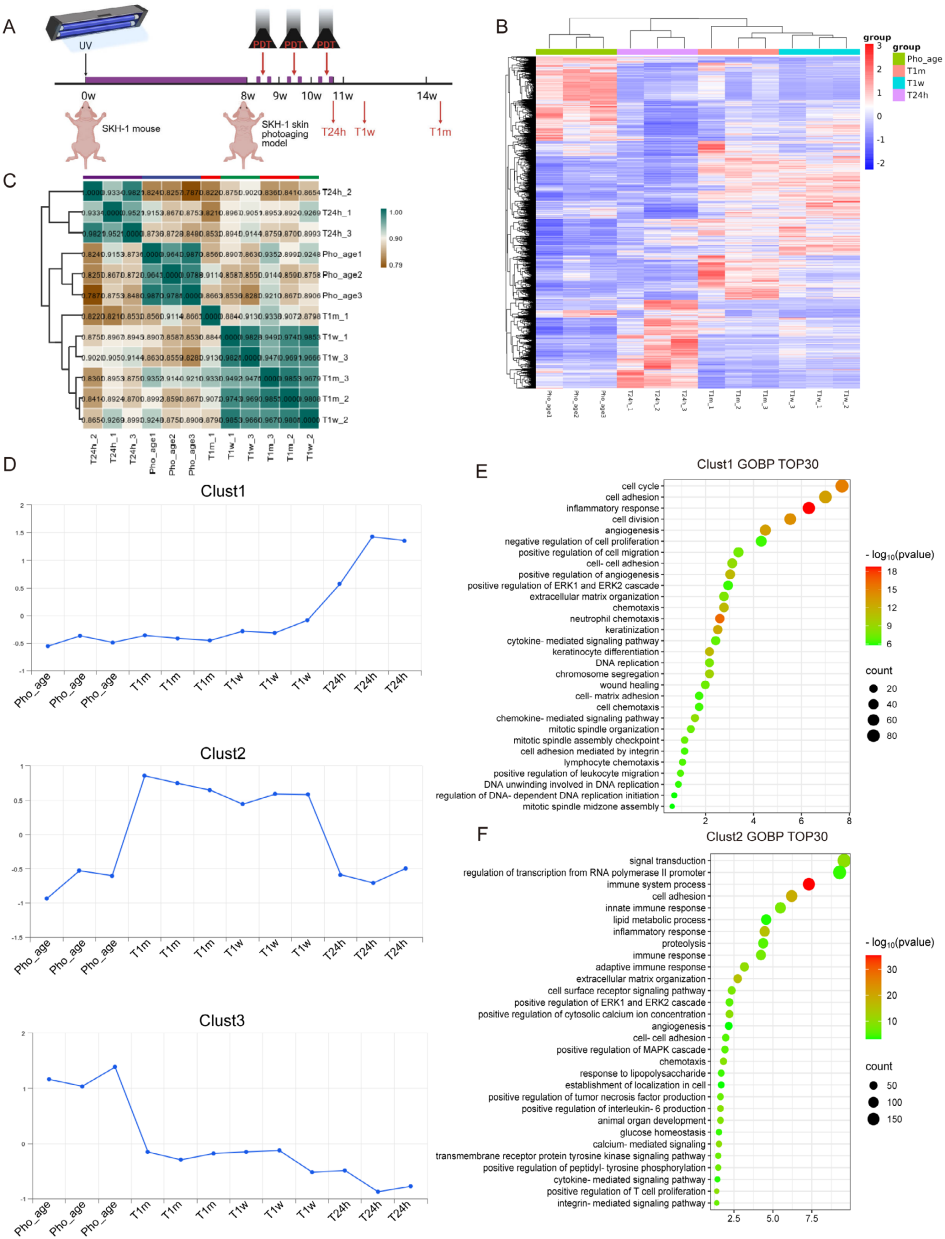
Transcriptome profiling reveals that ALA‐PDT has different effects in the early and late stages. (A) Diagram of the experimental paradigm. Mouse dorsal skin was acquired 24 h (T24h), 1 week (T1w), and 1 month (T1m) after ALA‐PDT for further study. (B) Heatmap of DEGs in different groups. (C) Correlation analysis between different groups. (D) Series test of cluster among different groups. (E, F) GO functional enrichment analysis of clust1 and 2, respectively.

SKH‐1 hairless mice were utilized to establish a photoaging model, subsequent to which they underwent weekly treatments with ALA‐PDT. Skin biopsy specimens were collected for analysis at three distinct time points: 24 h (T24h), 1 week (T1w), and 1 month (T1m) post‐final treatment, as illustrated in Figure 1A. The skin samples from the photoaged mice (Pho_age) used for sequencing were collected after more than 10 weeks, coinciding with the 24‐h time point after treatment in the ALA‐PDT group. ALA‐PDT treatment can significantly improve wrinkles caused by photoaging and maintain the improvement for at least 1 month (Figure S1A). Additionally, gross photographs, along with HE and Masson's trichrome staining, demonstrate that the photoaged phenotype in mice did not spontaneously resolve at the time point of the 14th week and beyond (corresponding to T1m) on the timeline (Figure S1C). Furthermore, significant improvements were recorded in both trans epidermal water loss (TEWL) and skin hydration 1 month after the treatment (Figure S1B). Histological assessment of the skin samples revealed a marked reduction in epidermal thickness following ALA‐PDT treatment, which had previously increased due to photoaging in the mice (Figure S1C). Additionally, Masson staining demonstrated a denser and more organized collagen fiber arrangement after ALA‐PDT treatment, indicating structural recovery in the dermal layer (Figure S1C).

In alignment with the findings reported in previous studies (Salminen et al. 2022; Gilchrest 2013), transcriptomic GSEA of skin tissues derived from photoaged mice versus normal control mice (aged 14–16 weeks) exhibited substantial reductions in functional categories encompassing lymphocyte activation, adaptive immune response, basement membrane, collagen composition, intercellular junctions, epidermal development and differentiation, as well as vascular and neurological development (Figure S2A). Notably, these functionalities were significantly enhanced 24 h following ALA‐PDT treatment and sustained for at least 1 month after treatment (Figures S2B–D and S3A, Table S1). Furthermore, we performed qPCR validation on partial genes in mouse tissues, as well as Western Blot (WB) validation for collagen changes (Figure S3B,C).

Proteomic analysis of skin lesions before and after ALA‐PDT treatment demonstrated a correlation with the transcriptomic outcomes (Figure S4A). Additionally, our findings revealed that ALA‐PDT significantly enhanced extracellular matrix components' expression, adaptive immune responses, angiogenesis, and intercellular adhesion functions (Figure S4B–D). These results further substantiate the therapeutic potential of ALA‐PDT in photoaging.

### 3.2 Transcriptome Profiling Reveals That ALA‐PDT Has Different Effects in the Early and Late Stages

Heatmap visualization and correlation analysis revealed that the skin samples collected at the early stage (24 h after ALA‐PDT) and late stages (1 week and 1 month after ALA‐PDT) represented distinct states (Figure 1B,C). To comprehensively investigate the functional alterations across these three states—photoaged skin, early post‐ALA‐PDT treatment, and late post‐ALA‐PDT treatment—we conducted a series of clustering analyses (Figure 1D). Our findings indicated that genes highly expressed in the early post‐ALA‐PDT stage were predominantly enriched in cell cycle‐related functions (Figure 1E). Additionally, cell division, DNA replication, and mitotic spindle midzone assembly were also among the enriched functions (Figure 1E), suggesting that ALA‐PDT may stimulate cell proliferation during the early phase. However, in the late post‐ALA‐PDT stage, genes with high expression were significantly enriched in immune response, cell adhesion, glucose metabolism, and lipid metabolism (Figure 1F).

### 3.3 ALA‐PDT Can Improve Cellular Senescence in the Early Stage

**FIGURE 2:**
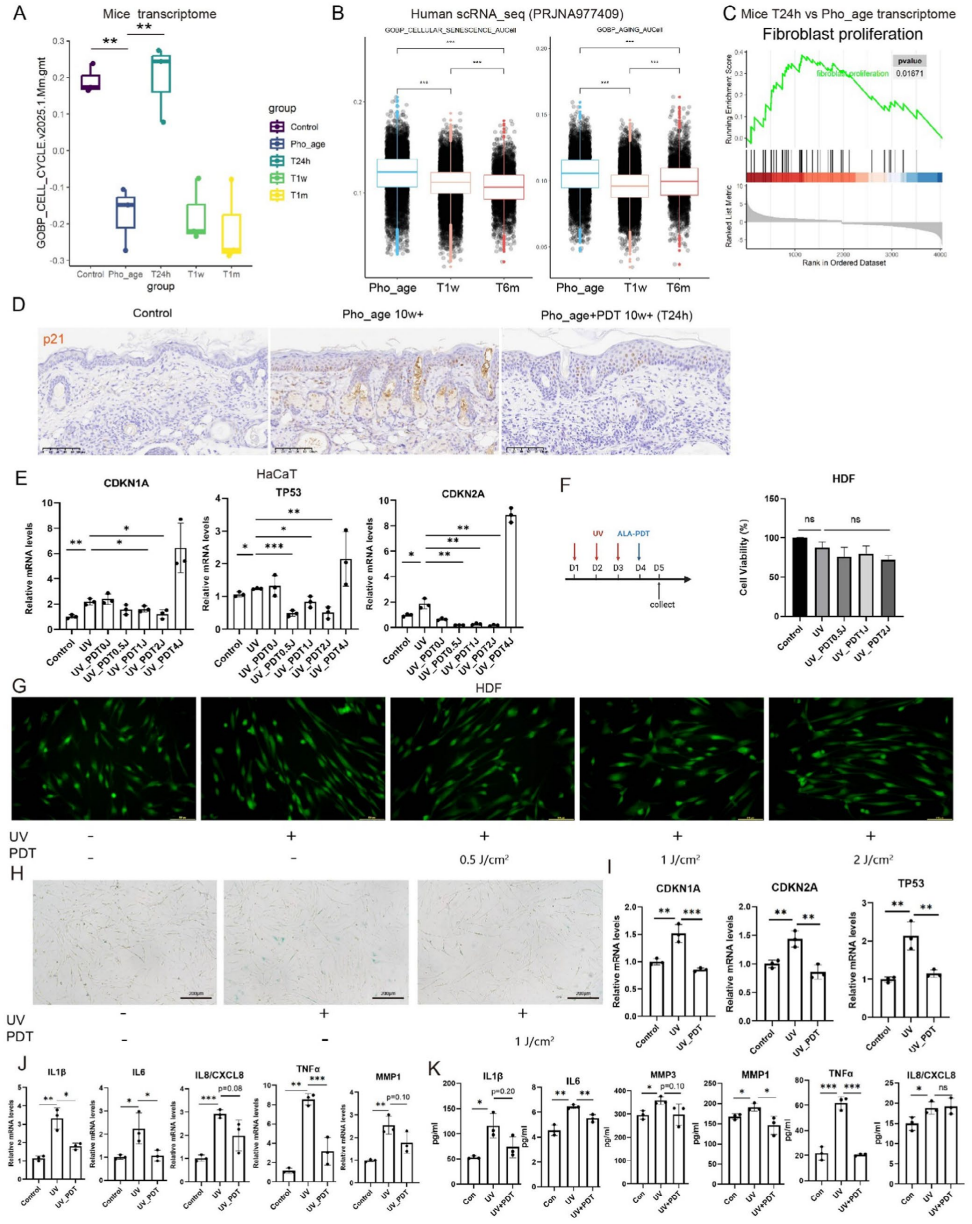
ALA‐PDT can improve cellular senescence in the early stage. (A) Cell cycle gene set variation analysis (GSVA) in different groups. (B) Boxplots diagrams of AUCell scores of GO pathway in keratinocyte in single‐cell sequencing data from photoaged skin in human (PRJNA977409). (C) GSEA analysis of DEGs between T24h and photoaged skin (Pho_age) (*p* < 0.05). (D) Representative p21‐stained sections of mice skin tissues. (E) The mRNA levels of cellular senescence‐related genes in HaCaT cells. (F) Schematic diagram of HDF cell treatment (left), CCK8 assay (right). (G) Representative images of live‐dead staining of HDF cells. (H) Representative SA‐β‐Gal staining of HDF cells. (I, J) mRNA level changes of cellular senescence‐associated genes (I) and SASP (J) in HDF cells. (K) ELISA assay for SASP in HDF cell supernatant. *p < 0.05, **p < 0.01, ***p < 0.001.

Given that cellular senescence is recognized as a pivotal driver of skin photoaging (Fitsiou et al. 2021), and our prior studies have demonstrated ALA‐PDT's capacity to stimulate the cell cycle (Figure 1E, Figure S3A), we aimed to look further into the potential of ALA‐PDT to ameliorate cellular senescence. Initially, Gene Set Variation Analysis (GSVA) of transcriptomic data further confirmed the significant upregulation of cell cycle functions following ALA‐PDT treatment (Figure 2A). Additionally, GSEA revealed a notable upregulation of fibroblast proliferation function 24 h after ALA‐PDT treatment (Figure 2C). Furthermore, analysis of previously published single‐cell sequencing data from human photoaged skin (PRJNA977409) indicated that ALA‐PDT treatment led to a significant downregulation of cellular senescence and aging functions in keratinocytes (Figure 2B).

Immunostaining for p21, p16, lamin B1, and ki67 (markers for cellular senescence) in the skin of photoaging mice demonstrated a reduction in the number of senescent cells after PDT treatment (Figure 2D, Figure S5A). There have been some in vitro studies in the past examining how higher doses of ALA‐PDT directly induce senescent cell death (Grigalavicius et al. 2016; Zhou et al. 2016; Ji et al. 2010). However, the in vivo scenario tends to be more complex, as not all cells are exposed to high‐dose photodynamic effects. As the depth from the surface increases, the effective dose of both drugs and light decreases. Consequently, we aim to explore other effects of lower‐dose ALA‐PDT on senescent cells. Exposure of HaCaT cells to UV radiation for 1 day (100 mJ/cm^2^) induced an increase in CDKN1A, CDKN2A, and TP53 mRNA levels, which was improved by low‐dose ALA‐PDT (0.5‐2 J/cm^2^). However, a higher dose of ALA‐PDT (4 J/cm^2^) exacerbated their expression (Figure 2E). To better simulate chronic photoaging in vitro, we employed three consecutive days of UV (100 mJ/cm^2^) irradiation on HDF cells, followed by photodynamic treatment at different doses 24 h later (Figure 2F, left). Both live‐dead staining and CCK8 assays confirmed that ALA‐PDT at doses of 0.5–2 J/cm^2^ does not induce death in senescent HDF cells (Figure 2F,G). Furthermore, SA‐β‐Gal staining and qPCR analysis of CDKN1A, CDKN2A, and TP53 demonstrated that 1 J/cm^2^ ALA‐PDT ameliorates cellular senescence (Figure 2H,I). Consistently, both mRNA level analysis of HDF cells and ELISA assays of HDF cell supernatant indicated that 1 J/cm^2^ ALA‐PDT reduces the expression and secretion of senescence‐associated secretory phenotype (SASP) factors (Figure 2J,K). We exposed normal human epidermal keratinocytes (NHEK) to 100 mJ/cm^2^ UV irradiation for three consecutive days, followed by treatment with different doses of ALA‐PDT. Our findings similarly revealed that lower doses of ALA‐PDT effectively ameliorate cellular senescence (Figure S5B).

### 3.4 Multi‐Omics Analysis Revealed That ALA‐PDT Significantly Affected Glucose Metabolism

**FIGURE 3:**
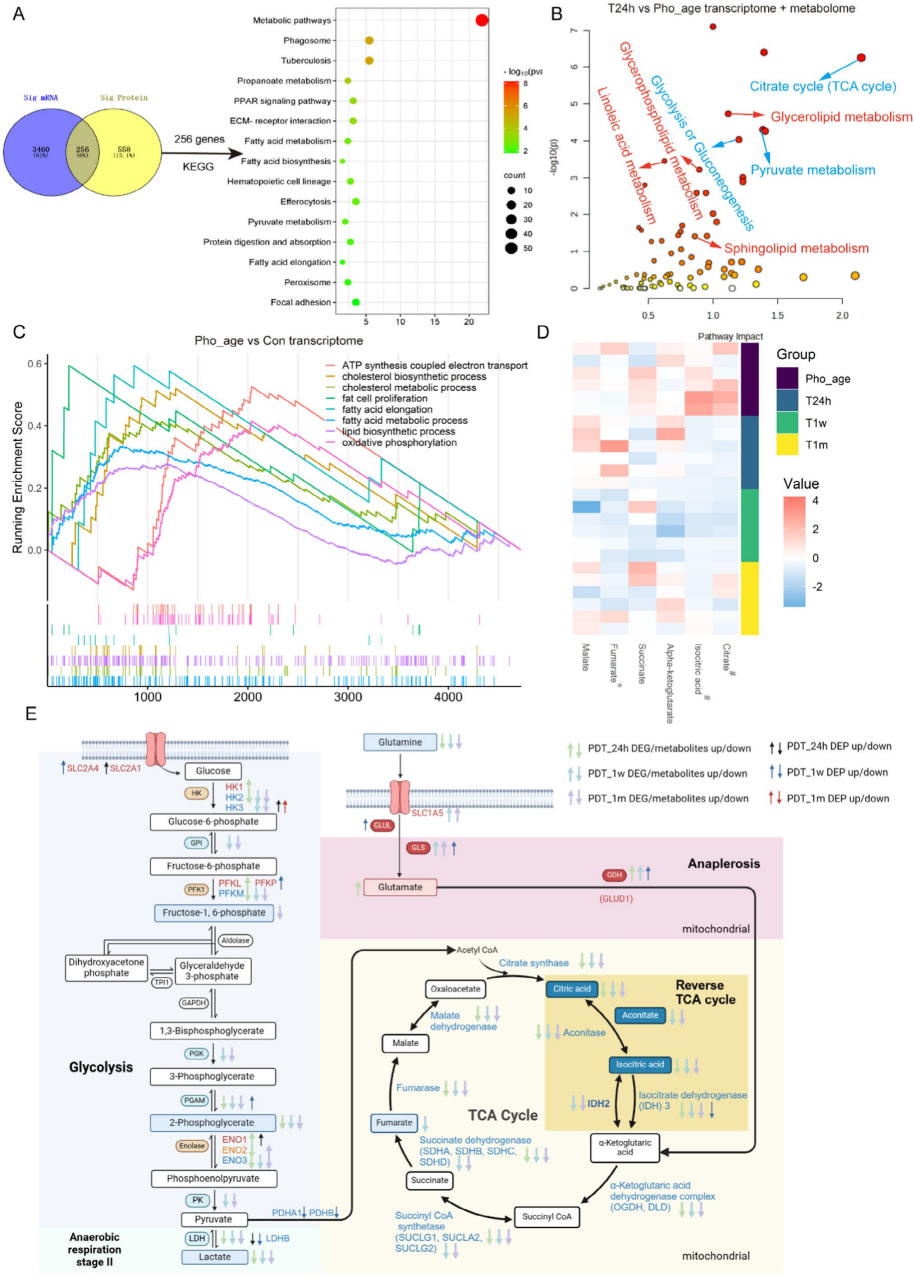
Multi‐omics analysis revealed that ALA‐PDT significantly affected glucose metabolism. (A) The differentially expressed genes and proteins between T24h and photoaged skin were intersected, and these 256 genes were further analyzed for KEGG functional enrichment. (B) Combined analysis of transcriptome and metabolome performed by MetaboAnalyst. (C) GSEA analysis of DEGs between photoaged and control mice skin (14–16 weeks of age). (D) Heatmap of TCA‐related metabolites. ^#^Metabolite was significantly downregulated at 24 h, 1 week, and 1 month after ALA‐PDT treatment. *Metabolite was significantly downregulated at 1 week after ALA‐PDT treatment. (E) Schematic diagram of changes in enzymes and metabolites associated with glucose metabolism. ↑↓*p* < 0.05.

To further investigate the mechanism underlying the improvement of cellular senescence by ALA‐PDT, we intersected the differentially expressed genes identified from transcriptome sequencing and proteomic profiling at 24 h after ALA‐PDT treatment. We then conducted functional enrichment analysis on the 256 genes obtained from this intersection. The results revealed that metabolic pathways were the most significantly enriched functions (Figure 3A). Therefore, we further performed metabolomic analysis of the skin lesions and combined analysis with transcriptome analysis. Our findings revealed that the DEGs and differential metabolites were prominently enriched in glucose and lipid metabolism pathways, including the tricarboxylic acid (TCA) cycle, glycolysis and gluconeogenesis, pyruvate metabolism, glycerolipid metabolism, and glycerophospholipid metabolism. These enrichments persisted for at least 1 month after ALA‐PDT treatment (Figure 3B; Figure S6A,B). Concordantly, transcriptomic analysis demonstrated a significant upregulation of functions related to glucose and lipid metabolism in photoaging skin, such as ATP synthesis coupled with electron transport, oxidative phosphorylation, fatty acid elongation, and lipid biosynthetic processes. Notably, these alterations could be ameliorated by ALA‐PDT (Figure S6C–H). Furthermore, heatmaps derived from transcriptomic and proteomic data indicated a decrease in the expression of most glucose metabolism‐related genes following ALA‐PDT treatment, particularly at 1 week after treatment (Figures S7 and S8). Since the TCA cycle was the most significantly enriched, we further examined the changes in its specific metabolites and found that CA and isocitrate were significantly down‐regulated after ALA‐PDT treatment, and this downregulation persisted for at least 1 month (Figure 3D).

To present the changes in glucose metabolism in a more intuitive manner, we have displayed proteins and molecules associated with glucose metabolism in Figure 5E. Within this figure, distinct arrows are employed to signify significant alterations in gene expression levels at both the RNA and protein levels, as well as notable variations in the concentrations of small molecule metabolites. Our analysis revealed that the expression of the majority of metabolites and related enzymes involved in both anaerobic respiration and the tricarboxylic acid (TCA) cycle was markedly decreased following ALA‐PDT treatment (Figure 3E). Conversely, the metabolites and enzymes of the glutamate anaplerotic pathway were significantly upregulated (Figure 3E). These findings suggest that ALA‐PDT may compensate for energy needs through the glutamate anaplerotic pathway.

It is important to note that these omics data were derived from a single skin tissue sample and cannot represent single‐cell variations; therefore, we conducted in vitro experiments for validation. Following three consecutive days of 100 mJ/cm^2^ UV irradiation, we applied 1 J/cm^2^ ALA‐PDT treatment on the fourth day and immediately sent the samples for Seahorse analysis. The results showed that the UV‐treated group exhibited significantly elevated extracellular acidification rate (ECAR) compared to the normal control group, while ALA‐PDT treatment reduced ECAR to a certain extent. Conversely, the oxygen consumption rate (OCR) in the UV group was lower than that in the normal group, and the ALA‐PDT‐treated group demonstrated even lower OCR levels (Figure S9).

### 3.5 ALA‐PDT Ameliorated Cellular Senescence by Reducing the Citrate Content

**FIGURE 4:**
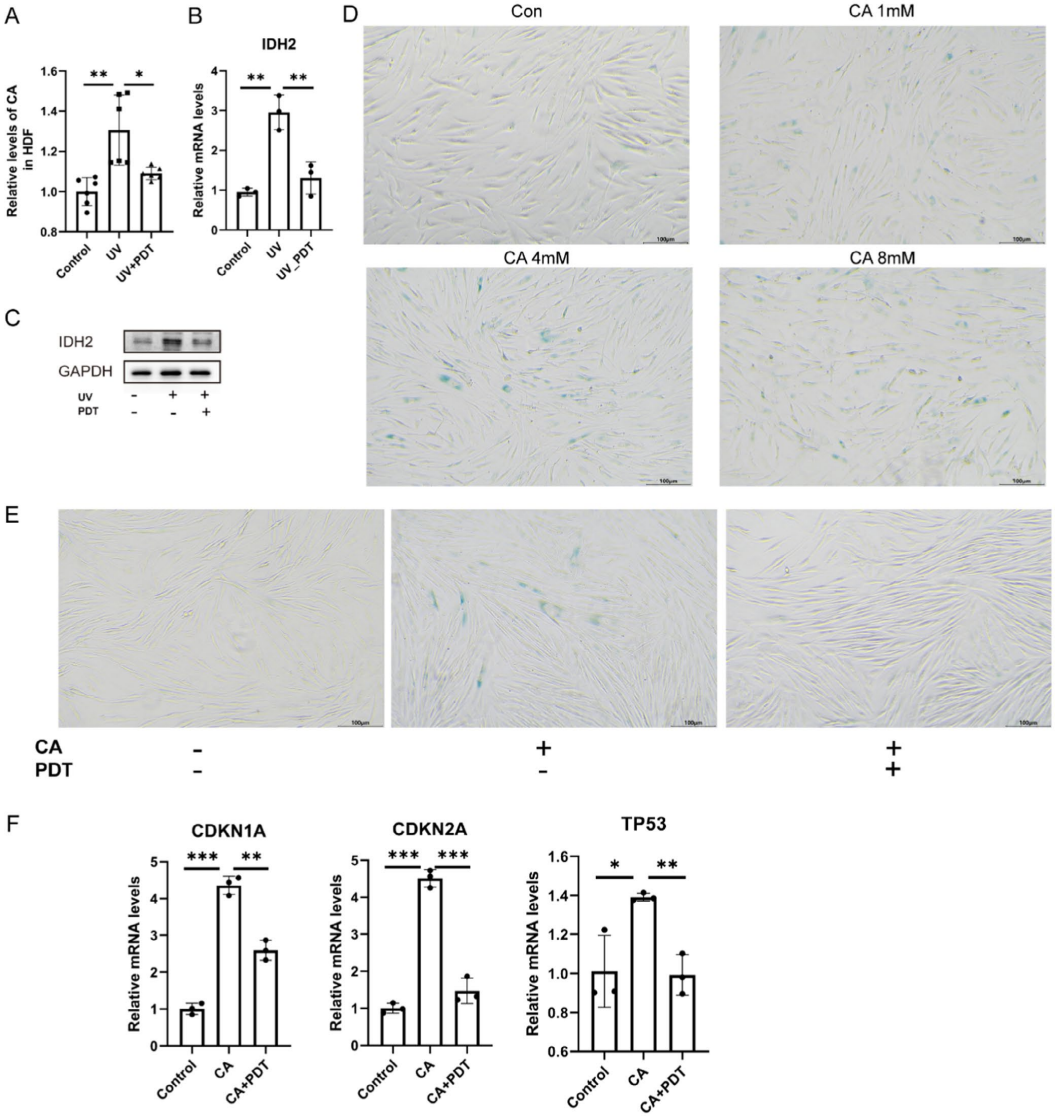
ALA‐PDT ameliorated cellular senescence by reducing the citric acid content. (A) Relative levels of CA in HDF cells. (B) The mRNA levels of IDH2 in HDF. (C) The protein levels of IDH2 in HDF. (D) Representative SA‐β‐gal staining of HDF cells after treatment with different concentrations of CA for 24 h. (E) Representative SA‐β‐gal staining of HDF cells after 24 h of 4 mM CA treatment. (F) The mRNA levels of cellular senescence‐related genes in HDF cells after 24 h of 4 mM CA treatment and 24 h after 1 J/cm^2^ ALA‐PDT treatment. *p < 0.05, **p < 0.01, ***p < 0.001.

Given that both the DEGs from transcriptome sequencing analysis and the differential metabolites from metabolome analysis were significantly enriched in the TCA cycle (Figure 3B, Figure S6A,B), and that the expression levels of TCA‐related proteins in proteome analysis were also significantly downregulated, which is consistent with the results of transcriptome sequencing analysis, we focused our attention on the TCA cycle. In addition, since CA is the upstream differential metabolite of the TCA cycle, we speculate that CA may be the key to ameliorating cellular senescence in ALA‐PDT therapy, and in vitro studies were done. After 3 consecutive days of UV‐induced cellular senescence on HDF, treatment with 1 J/cm^2^ ALA‐PDT found that UV resulted in a significant increase in citric acid content, which can be ameliorated by ALA‐PDT (Figure 4A). Consistent with transcriptomic sequencing results (Figure 3E), cell experiments demonstrated that the expression of IDH_2_, a key enzyme in the reverse TCA (rTCA) cycle, increased after UV irradiation, and PDT could restore it (Figure 4B,C). This suggested that ALA‐PDT may reduce citric acid content by inhibiting the rTCA. We further investigated the role of CA itself and found that the addition of CA alone can cause cellular senescence through SA‐β‐Gal staining (Figure 4D), while ALA‐PDT can ameliorate 4 mM CA‐induced cellular senescence (Figure 4E,F). These results suggest that UV‐induced senescence also involves a metabolic shift leading to CA accumulation and rTCA activation, and that ALA‐PDT can prevent UV‐induced senescence by counteracting this metabolic reprogramming.

### 3.6 Mitohormesis Was Triggered by ALA‐PDT Validated by Multi‐Omics and In Vitro Experiments

**FIGURE 5:**
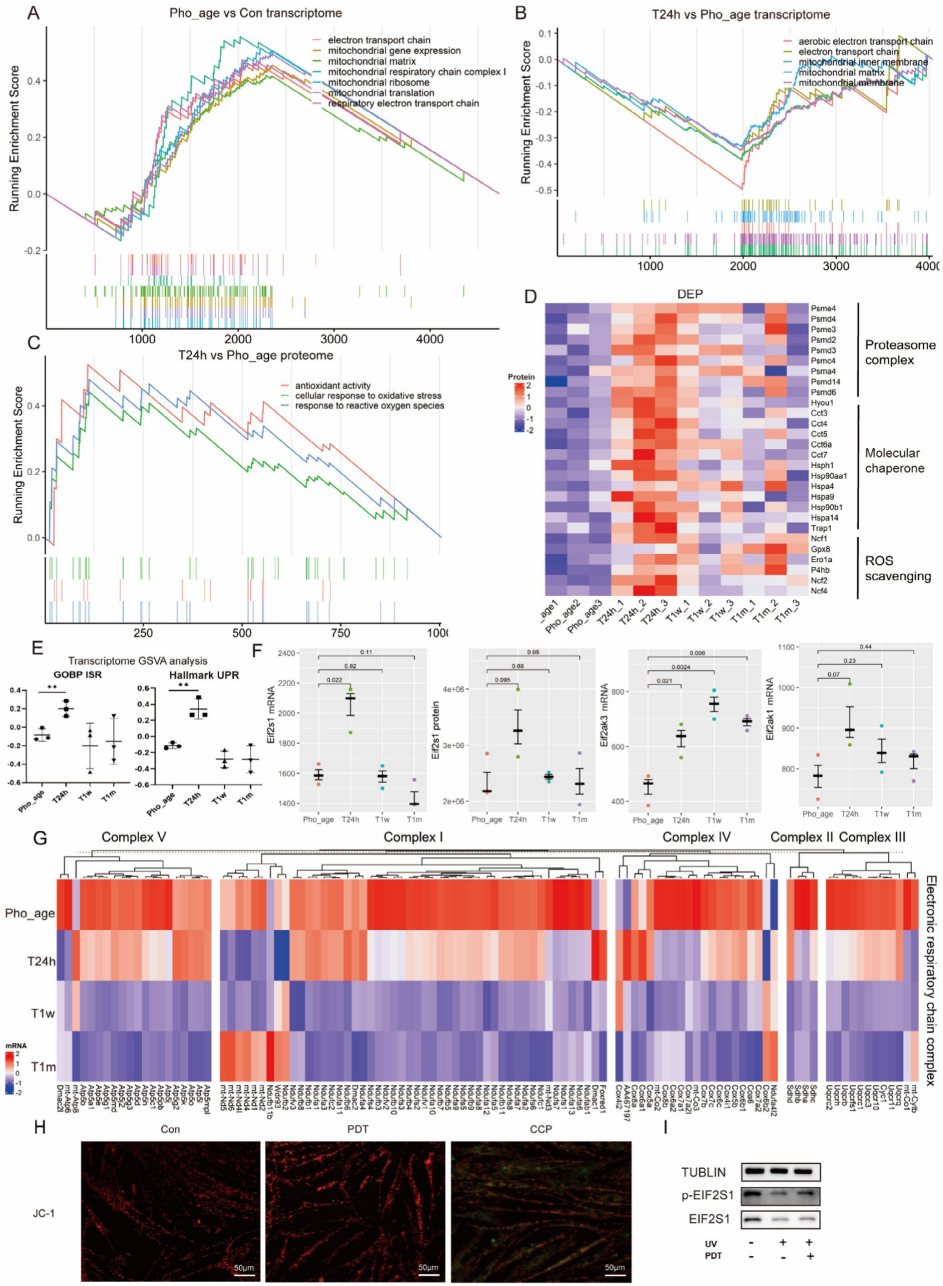
Mitohormesis was triggered by ALA‐PDT validated by multi‐omics and in vitro experiments. (A, B) GSEA analysis of DEGs in different groups (*p* < 0.05). (C) GSEA analysis of DEPs in different groups (*p* < 0.05). (D) Heatmap of DEPs in different groups. (E) GSVA of GO and hallmark pathways in transcriptomics. (F) Relative gene expression levels in transcriptomics and proteomics. (G) Heatmap of electronic respiratory chain complex‐related genes in different groups in transcriptomics. (H) JC‐1 assay of 1 J/cm^2^ ALA‐PDT treatment in HDF. (I) The protein levels of EIF2S1 and p‐EIF2S1 in HDF. *p < 0.05, **p < 0.01, ***p < 0.001.

Since ALA‐PDT acts by generating ROS such as single oxygen species and oxygen radicals in mitochondria (Ji et al. 2006), and the TCA cycle also occurs in mitochondria, we further focus on the changes of ALA‐PDT to mitochondria. Transcriptomics showed that the functions of the electron transport chain, inner mitochondrial membrane, and mitochondrial matrix were significantly down‐regulated after ALA‐PDT treatment and maintained at least 1 month after treatment (Figure 5B, Figure S10A), and photoaging significantly increases these functions compared to healthy mice skin (Figure 5A). Interestingly, proteomics found that antioxidant activity and oxidative stress function were significantly upregulated after ALA‐PDT treatment (Figure 5C, Figure S10B).

These results suggest that low‐dose ALA‐PDT may induce mitohormesis, which is defined as low levels of free radicals in the mitochondria that trigger an appropriate stress response to exert a protective role. Mitohormesis has some characteristics: (1) ROS resistance, (2) increased expression of chaperones, proteasome complex, and HSF_1_ target genes, and (3) mitochondrial aerobic respiration inhibition, which was identified previously (Boos et al. 2019; Timblin et al. 2021). Further analysis of DEPs revealed evidence that ALA‐PDT significantly increased the levels of proteins involved in ROS scavenging, molecular chaperones, and proteasome (Figure 5D), and genes targeted by HSF1 (Figure S11C). Similar findings were made in the analysis of DEGs (Figure S11A,B). In addition, mitohormesis works by initiating the integrated stress response (ISR) and unfolded protein response (UPR) of mitochondria (Anderson and Haynes 2020). GSVA analysis of the transcriptome and proteome also found that ALA‐PDT could upregulate both functions (Figure 5E, Figure S10C). The phosphorylation of Eif2s1, a key gene of mitohormesis (Cheng et al. 2023), and the expression of itself and its kinase were also significantly upregulated by ALA‐PDT (Figure 5F,I). And consistent with the inhibition of mitochondrial aerobic respiration, the expression of the mitochondrial inner membrane electron respiratory transport chain complex was significantly reduced after ALA‐PDT treatment (Figure 5G). However, although ALA‐PDT can induce the above series of stress responses, ALA‐PDT treatment with a dose of 1 J/cm^2^ on HDF cells did not damage the membrane potential of mitochondria (Figure 5H), which also corresponded to the low‐dose damage in mitohormesis.

### 3.7 ALA‐PDT Triggered Mitohormesis to Reduce Citrate Content, Thereby Improving Cellular Senescence

**FIGURE 6:**
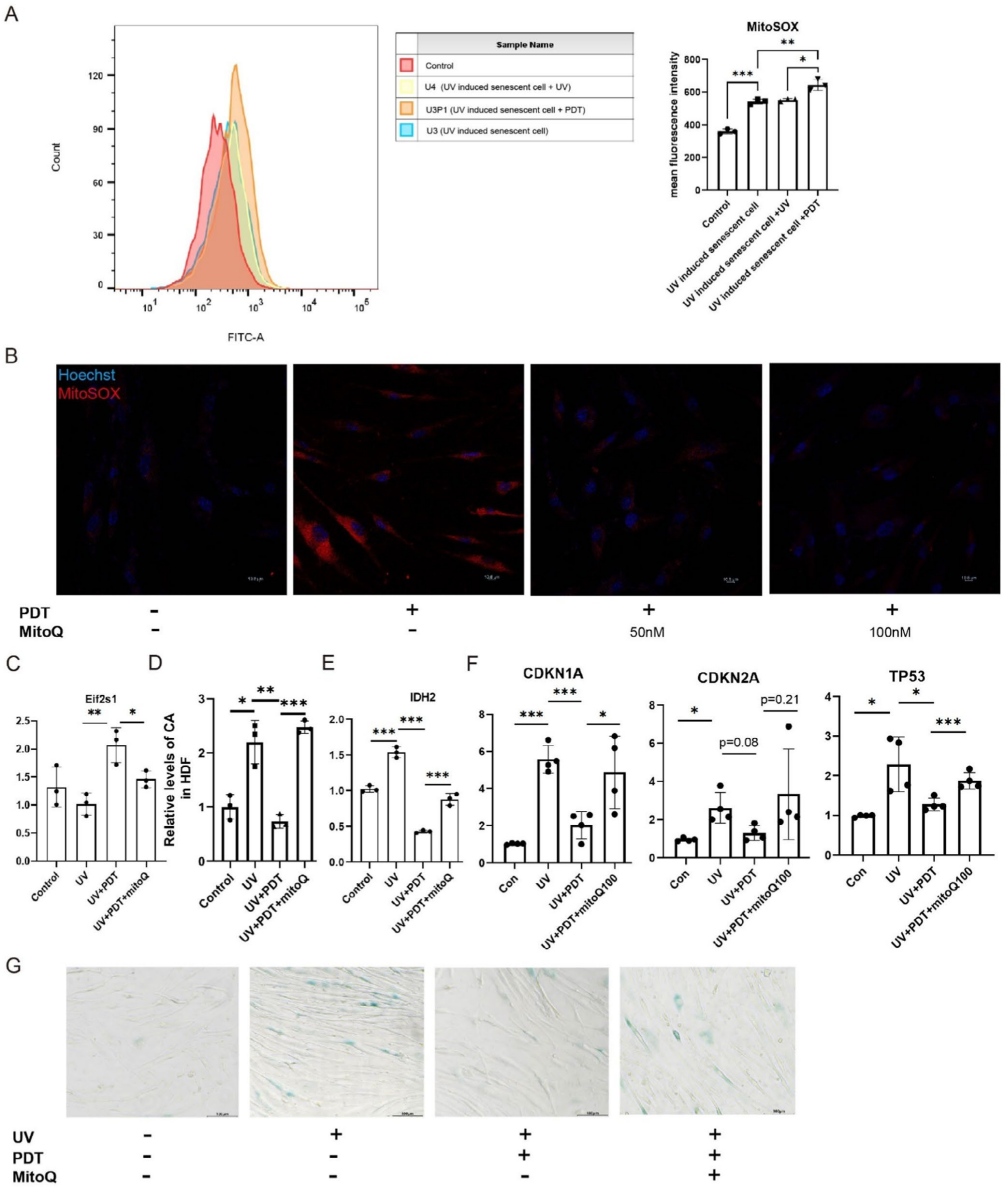
ALA‐PDT triggered mitohormesis to reduce citrate content, thereby improving cellular senescence. (A) MitoSOX Green Assay and Flow Cytometry Quantitative Analysis of HDF Cells Under Different Treatment Regimens. Control: No UV or ALA‐PDT treatment applied. U3: Consecutive 3‐day UV exposure (100 mJ/cm^2^ every 24 h), with samples collected 24 h after the final UV treatment. U4: Consecutive 4‐day UV exposure (100 mJ/cm^2^ every 24 h), with samples collected immediately after the final UV treatment. U3P1: Consecutive 3‐day UV exposure (100 mJ/cm^2^ every 24 h), followed by 1 J/cm^2^ ALA‐PDT treatment 24 h after the final UV exposure; samples collected immediately post‐PDT treatment. (B) Representative fluorescence photographs of live HDF cells MitoSox and Hoechst staining. (C) The mRNA levels of EIF2S1 in HDF cells. (D) Relative levels of CA in HDF cells. (E, F) The mRNA levels of IDH2, CDKN1A, CDKN2A, and TP53 in HDF cells. (G) Representative SA‐β‐gal staining of HDF cells. *p < 0.05, **p < 0.01, ***p < 0.001.

As previously described, HDF were exposed to UV irradiation for three consecutive days. On Day 4, they were treated with either 100 mJ/cm^2^ UV or 1 J/cm^2^ ALA‐PDT. Immediately post‐treatment, the MitoSOX Green assay was performed to detect mitochondrial ROS, followed by flow cytometry analysis. Results revealed that the 100 mJ/cm^2^ UV treatment did not induce significant mitochondrial ROS production at the immediate time point (U4 vs. U3). However, 24 h after three consecutive days of UV exposure, mitochondrial ROS levels were significantly elevated compared to normal controls (U3 vs. Con), suggesting that UV‐induced mitochondrial ROS generation is a delayed response. Consistent with the immediate inhibition of the oxygen consumption rate (OCR) by ALA‐PDT, the 1 J/cm^2^ ALA‐PDT treatment promptly enhanced mitochondrial ROS production (U3P1 vs. U3) (Figure 6A). Since it has been hypothesized that low‐dose ALA‐PDT induces mitohormesis by producing ROS in the mitochondria, we specifically antagonized ALA‐PDT‐generated mitochondrial ROS levels with mitoquinone mesylate (mitoQ). By the MitoSOX Red assay, 100 nM mitoQ was determined to be the optimal dose, which could effectively antagonize the mitochondrial ROS production of ALA‐PDT, thereby inhibiting the occurrence of mitohormesis (Figure 6B). In conformity, the expression of EIF2S1, the marker gene of mitohormesis, was up‐regulated in the model of UV‐induced cellular senescence after ALA‐PDT treatment, and the upregulated portion of ALA‐PDT was restored after treatment with mitoQ (Figure 6C). Similar findings were made in CA and IDH_2_, the reverse citric acid cycle marker gene (Figure 6D,E). Finally, we demonstrated that 100 nM mitoQ can antagonize the anti‐UVR‐induced cellular senescence effect of ALA‐PDT by SA‐β‐gal and qPCR assays (Figure 6F,G).

## 4 Discussion

The findings of this study elucidate the multifaceted mechanisms through which ALA‐PDT exerts its beneficial effects on skin photoaging, particularly highlighting its role in improving cellular senescence via the modulation of citrate levels through a process termed mitohormesis. The results indicate that PDT not only improves skin appearance by reducing wrinkles and enhancing hydration but also initiates profound biochemical changes at the cellular level that contribute to skin rejuvenation.

Our study found that ALA‐PDT not only improved skin photoaging from gross phenotype and histopathology, but also had a long‐lasting skin rejuvenation effect at the transcriptomic and proteomic levels. Clinically, transient acute inflammatory reactions such as dry skin, erythema, pustules, and edema occur within 24 h of ALA‐PDT treatment, accompanied by symptoms of burning, stinging, and itching, which usually resolve within 2–3 days (Wang et al. 2023). Similar to the clinical presentation, transcriptomic sequencing also found that ALA‐PDT caused different changes in the early (24 h) and late (1 week and 1 month) stages.

In the process of photoaging, UVR not only directly causes photochemical damage to nucleic acids and proteins, but also indirectly leads to aging through the production of ROS (Kammeyer and Luiten 2015). Previous studies have also found that ROS removal can slow down or improve photoaging in mice (Li, Wan, et al. 2024; Bang et al. 2021). However, ALA‐PDT has been recognized to exert a photodynamic effect through mitochondrial production of ROS, and ALA‐PDT has also been widely recognized in clinical practice to improve photoaging (Shi et al. 2021). This seems to contradict the fact that UVR causes aging through the production of ROS. Mitohormesis is able to explain the phenomenon that this low dose of mitochondrial stress can ameliorate cellular senescence. There have been some in vitro studies demonstrating that low‐dose ALA‐PDT is able to improve UVR‐induced cellular senescence, possibly through Nrf_2_, BER, and Bach_2_ signaling (Wang et al. 2020, 2024; Chen et al. 2019).

There have been many studies that have found that mitohormesis has a beneficial effect on the body through low toxicity. In many species, it has been found that mitochondrial dysfunction is disrupted to promote longevity (Campisi et al. 2019), caloric restriction can also prolong life through mitohormesis (Li, Wang, et al. 2024), and exercise induces mitohormesis production in proopiomelanocortin (POMC) neurons of the hypothalamus, leading to obesity resistance (Kang et al. 2021). In macrophages, moderate mitochondrial stress leads to mitohormesis being able to impair mitochondrial oxidation to produce acetyl‐CoA, which exerts anti‐inflammatory effects (Timblin et al. 2021). Similarly, in this study, combined transcriptomics and proteomics analyses found that ALA‐PDT had the most significant effect on metabolism, and further metabolomic analysis was also enriched for glucose metabolism‐related pathways.

Mitochondrial dysfunction triggers transcriptional stress in the nucleus, regulating the expression of genes involved in protein folding, antioxidant stress response, and metabolism (Campisi et al. 2019). In yeast, nematodes, and mammalian cells, inhibition of mitochondrial respiration activates a conserved nuclear gene expression response known as the “retrograde response” (Kenyon 2010). This response upregulates corresponding genes to activate alternative energy‐generating pathways and cytoprotective pathways. In yeast and nematodes, specific mutations that suppress genes responsible for the overall or individual retrograde response can inhibit lifespan extension in these mutants (Kirchman et al. 1999; Cristina et al. 2009). Studies in nematode mutants revealed that inhibition of genes in the mitochondrial electron transport chain can activate the mitochondrial unfolded protein response (UPRmt), which is essential for lifespan extension (Durieux et al. 2011). Mitochondrial dysfunction in neurons can activate UPRmt in distal tissues such as the intestine, suggesting the presence of circulating metabolic regulators that act across multiple tissues (Durieux et al. 2011). Multiple factors regulate UPRmt, including the stress‐1‐related activating transcription factor ATFS‐1, the homeobox transcription factor DVE‐1, the ubiquitin‐like protein UBL‐5, the mitochondrial protease ClpP, and the mitochondrial inner membrane transporter HAF‐1 (Lin and Haynes 2016).

Additionally, recent studies highlight the p38 signaling pathway downstream of ribosome stress response (RSR) as a key driver of senescence, particularly SASP regulation (McKenney and Regot 2025). Both in vitro and in vivo studies confirm UVR induces RSR, characterized by ribosomal reduction (Sinha et al. 2024; Vind et al. 2024). Our single‐cell sequencing of human photoaged skin revealed ALA‐PDT significantly reduces ribosomal protein transcription (Yan et al. 2023). Since phosphorylated eIF2α (P‐eif2s1) inhibits RSR, our findings showing increased P‐eif2s1 levels suggest ALA‐PDT may directly improve cellular senescence by suppressing RSR. However, the relationship between mitohormesis and RSR requires further experimental investigation.

Studies have reported that changes in energy metabolism are associated with the aging process. With increasing age, lactate dehydrogenase (LDH) in peripheral blood mononuclear cells gradually increases (Ravera et al. 2019). Cellular replicative senescence also shows higher glycolytic activity and lactate production, enhanced expression of LDHA, increased TCA cycle activity, and mitochondrial respiration (Stabenow et al. 2022). Consistently, increased LDH and enhanced glycolytic activity were also found in UV‐induced cell senescence, and inhibition of glycolysis can delay cell senescence (Park et al. 2023). In this study, glycolysis‐related metabolites and key enzymes were also found to be reduced after ALA‐PDT treatment, suggesting that this may be one of the mechanisms by which ALA‐PDT leads to skin rejuvenation. However, studies have shown that extracellular CA accumulates after certain types of aging (Mycielska et al. 2022), and that exogenous citrate can induce senescence in tumor cells (Zhao et al. 2022). This study found that citrate content was significantly reduced after ALA‐PDT treatment, and in vitro experiments also verified that exogenous addition of citrate can induce cell senescence, and ALA‐PDT can improve it.

It is important to note that omics profiling captures variations in a single tissue sample. RNA‐seq revealed that photoaged skin exhibits elevated oxidative phosphorylation levels compared to normal skin, whereas ALA‐PDT treatment leads to a persistent downregulation of oxidative phosphorylation levels in photoaged skin. This may indicate metabolic restoration rather than persistent suppression in skin tissue. Seahorse assays demonstrated that ALA‐PDT treatment reduced both OCR and ECAR compared to the UV group. We propose that low‐dose ALA‐PDT imposes a mild metabolic stress on UV‐preconditioned cells, triggering an adaptive stress response characterized by global metabolic slowdown. This “metabolic deceleration” reduces overall energy expenditure and biosynthetic activities, enabling cells to conserve resources for survival and damage repair—consistent with the concept of mitohormesis. ALA‐PDT‐induced ROS temporarily suppress mitochondrial respiration to minimize further ROS generation via the electron transport chain, preventing oxidative damage cascades—a “trade‐off” strategy for survival. The SASP secretion process is energetically costly (requiring protein synthesis, folding, and exocytosis). By downregulating SASP, ALA‐PDT conserves cellular energy, reducing glycolytic demand (evidenced by ECAR reduction) and restoring metabolic flux toward homeostatic levels (approaching control group ECAR).

IDH_2_ has been shown to reduce and convert α‐ketoglutarate to citric acid, a metabolic pathway that increases in factors such as impaired mitochondrial respiration, hypoxic environments, or cancer cell metabolism, and is considered a key enzyme in the rTCA cycle (Heinz et al. 2022). However, the presence or absence of rTCA in senescent cells is still inconclusive, and this study found that IDH_2_ expression increased and CA content also increased during UV‐induced cellular senescence, suggesting the presence of rTCA, but further experimental verification is still needed.

## Author Contributions

Y.Y. and Q.C. established and treated photoaging mouse models, conducted sequencing data analysis, and drafted the initial manuscript. Y.W. and Q.Z. provided experimental technical guidance and support. Y.Z. and G.Y. assisted with sequencing data analysis and cellular experiment analysis. Z.C. and H.Z. revised and polished the manuscript and provided funding support. Q.Z., X.W., and P.W. conceived the study, revised the manuscript, and provided funding support.

## Funding

This research was supported by the National Natural Science Foundation of China (82303993), the Natural Science Foundation of Shanghai Municipality (22ZR1455400), the National Key Research and Development Program (2022YFC2504700 and 2022YFC2504705), and the Shanghai “Rising Stars of Medical Talents” Youth Development Program SHWSRS (2023)_062.

## Ethics Statement

Reviewed and approved by the Local Ethics Committee of Shanghai Skin Disease Hospital, Shanghai, China.

## Consent

The authors have nothing to report.

## Conflicts of Interest

The authors declare no conflicts of interest.

## Supporting information


**Figures S1–S11:** acel70328‐sup‐0001‐FiguresS1‐S11.docx.

**Table S1:** acel70328‐sup‐0002‐TableS1.xlsx.

## Data Availability

The data that support the findings of this study are available on request from the corresponding author. The data are not publicly available due to privacy or ethical restrictions.
