# Supplementary material for: Multi‐Omics Analysis Reveals Photodynamic Therapy Ameliorating Skin Photoaging by Improving Cellular Senescence Through Mitohormesis‐Mediated Reduction of Citrate Content
Source: Aging Cell. 2025 Dec 28;25(1):e70328. doi: 10.1111/acel.70328 (PMC12744960; doi:10.1111/acel.70328)
Supplement: Supplementary file 1 — Figures S1–S11: acel70328‐sup‐0001‐FiguresS1‐S11.docx. [file ACEL-25-e70328-s001.docx]

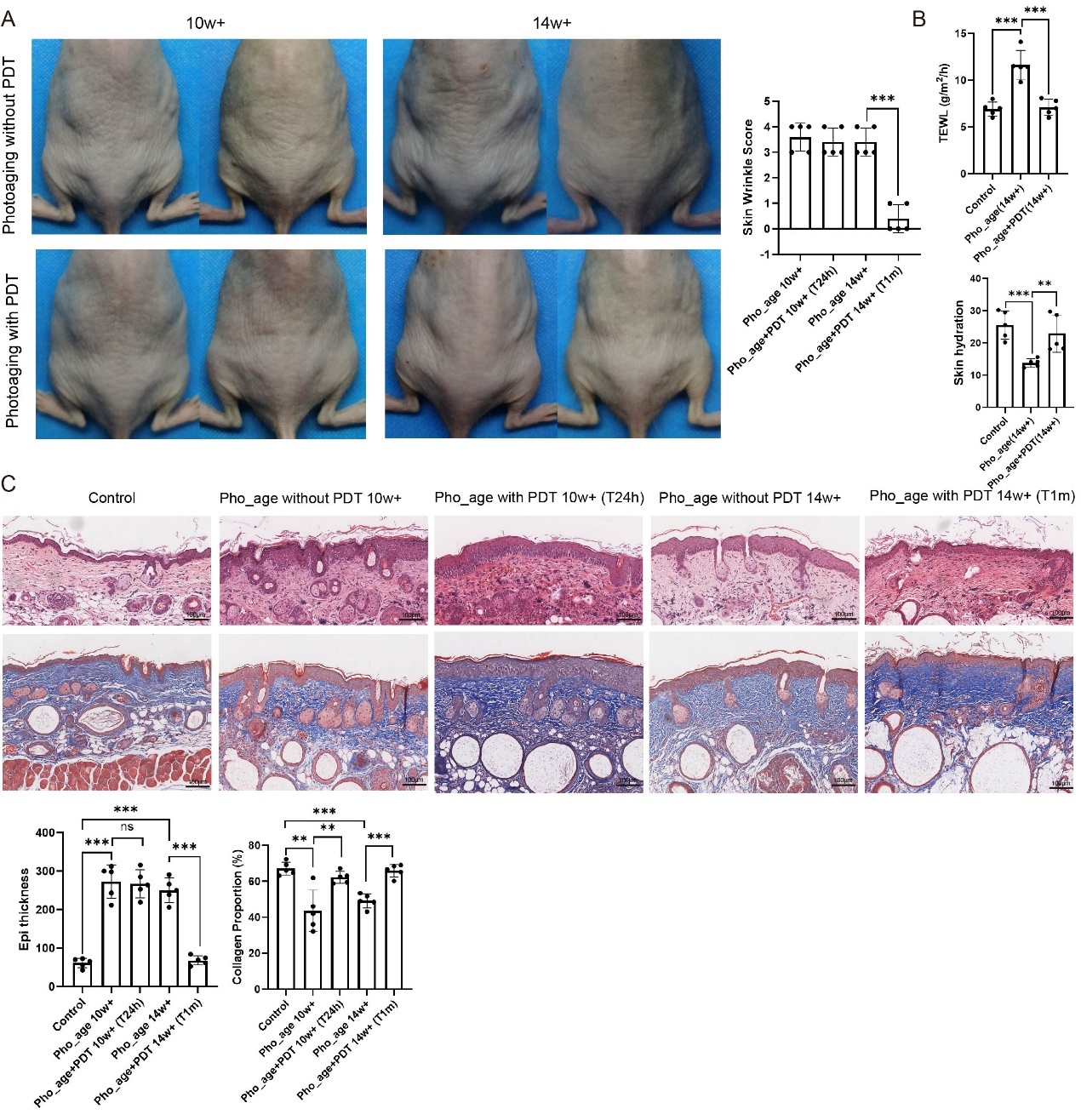


**Supplementary figure 1.** ALA-PDT attenuated photoaging in SKH-1 mice. A) Representative mouse images before and after treatment in the PDT/pho_age group. Skin wrinkles were scored according to previously established methods, using the following criteria: 0—Smooth skin; 1—Fine wrinkle; 2—A little bit shallow wrinkles; 3—Shallow wrinkles across the dorsal skin; 4—Deep and coarse wrinkles with laxity; 5—Increased wrinkle depth; 6—Severe wrinkles with skin damage occurs. B) Trans epidermal water loss (TEWL) and skin hydration of skin tissues (**n = 5** mice for each group). C) Representative HE-stained sections and Masson-stained sections of skin tissues. And the changes in epidermal thickness were calculated based on HE staining (bottom left). Masson's trichrome staining shows alterations in collagenous areas (bottom right). *P<0.05, **P<0.01, ***P<0.001.


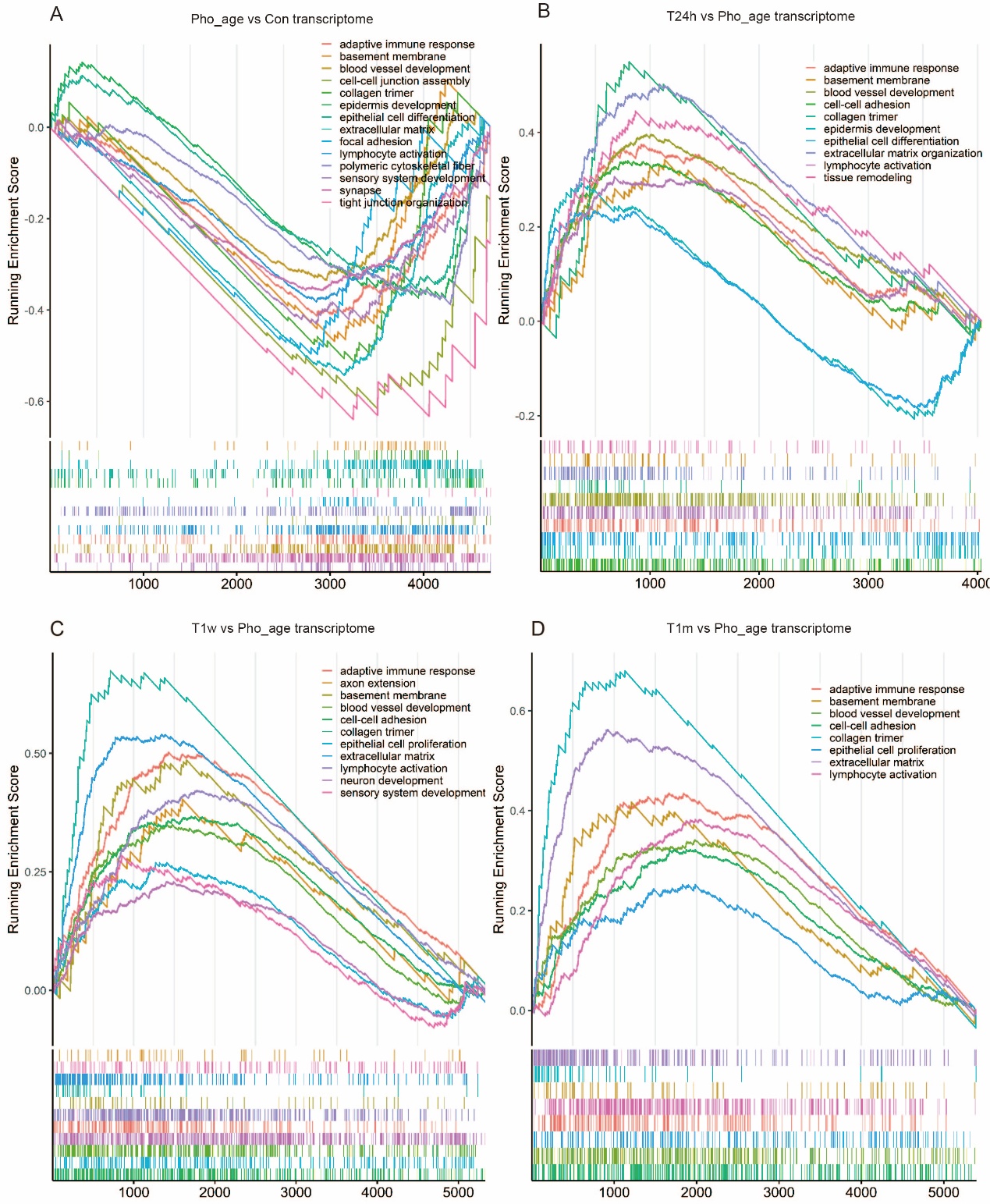
 **Supplementary figure 2**. ALA-PDT reversed multiple functional changes in photoaging in transcriptomics analysis. A-D) GSEA analysis of DEGs in different groups (P< 0.05).

**
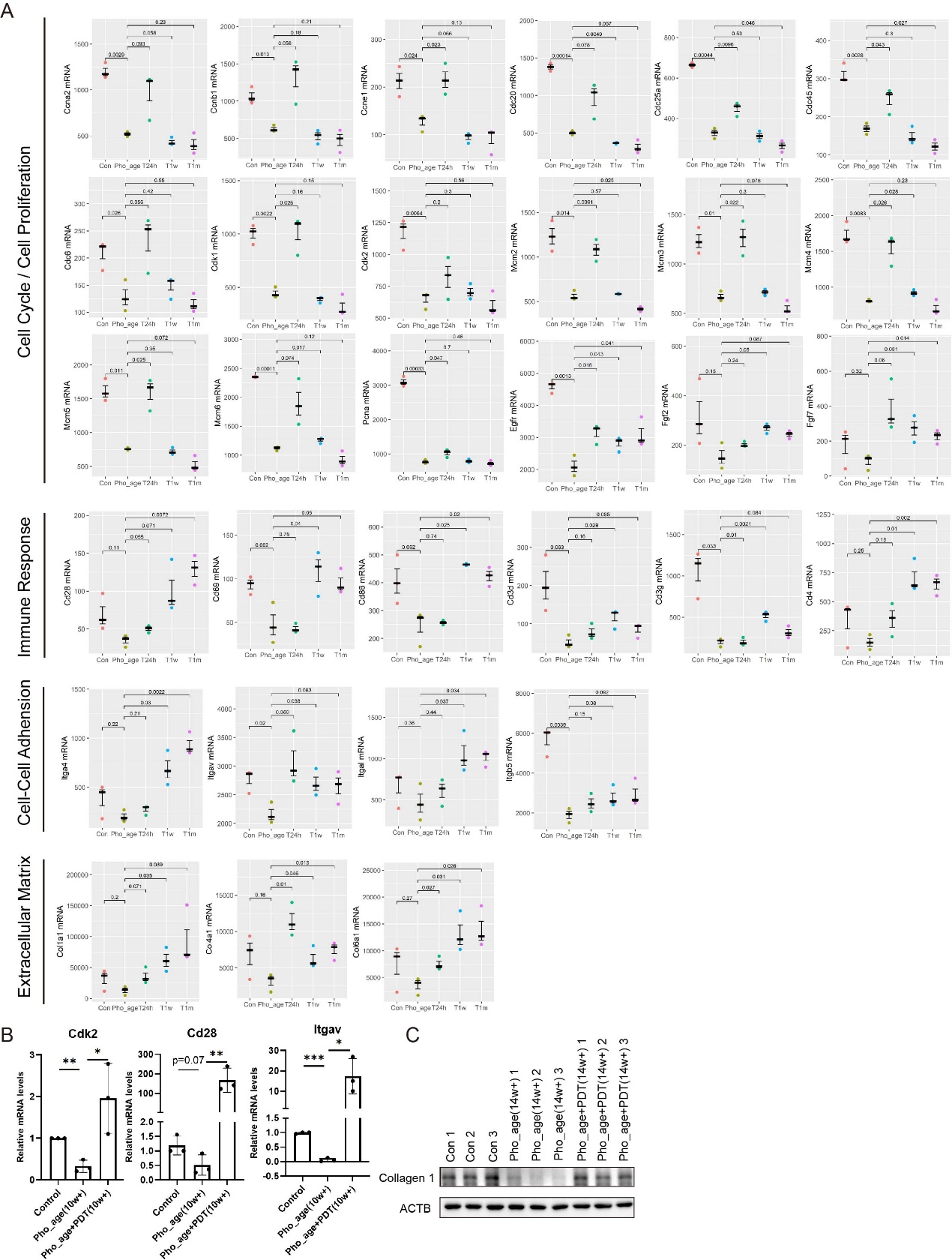
**

**Supplementary figure 3**. A) Transcriptomic Changes in Specific Gene Expression Associated with ALA-PDT-Mediated Reversal of Photoaging-Related Functions. B) qPCR Detection of Specific Genes in Mouse Skin Tissue. C) Western Blot (WB) Detection of Collagen in Mouse Skin Tissue. *P<0.05, **P<0.01, ***P<0.001.


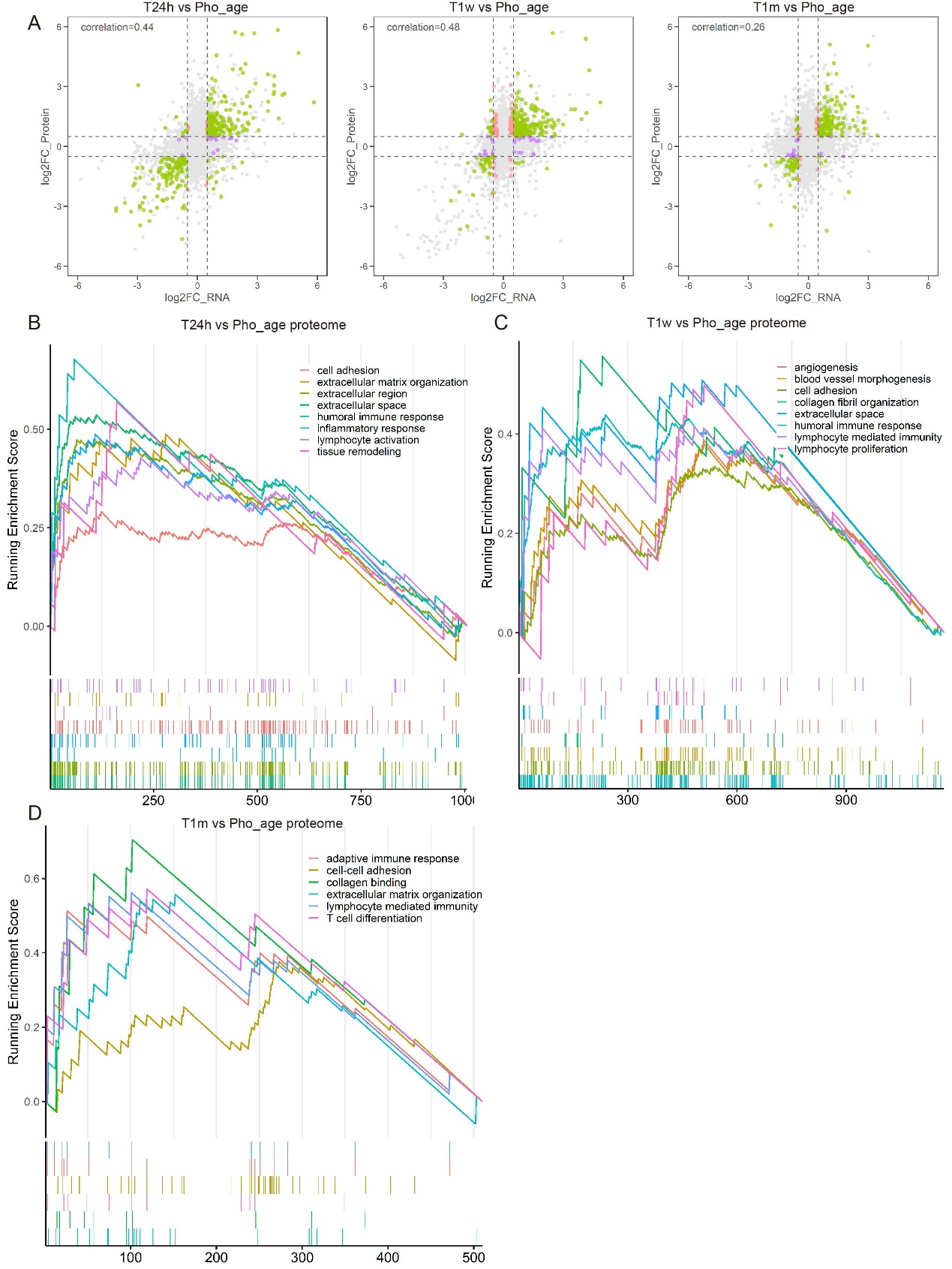
 **Supplementary figure 4.** A) Scatter plot of 9-quadrant associate analyses of mRNA and proteins from log2 FC. B-C) GSEA analysis of DEPs in different groups (P< 0.05).


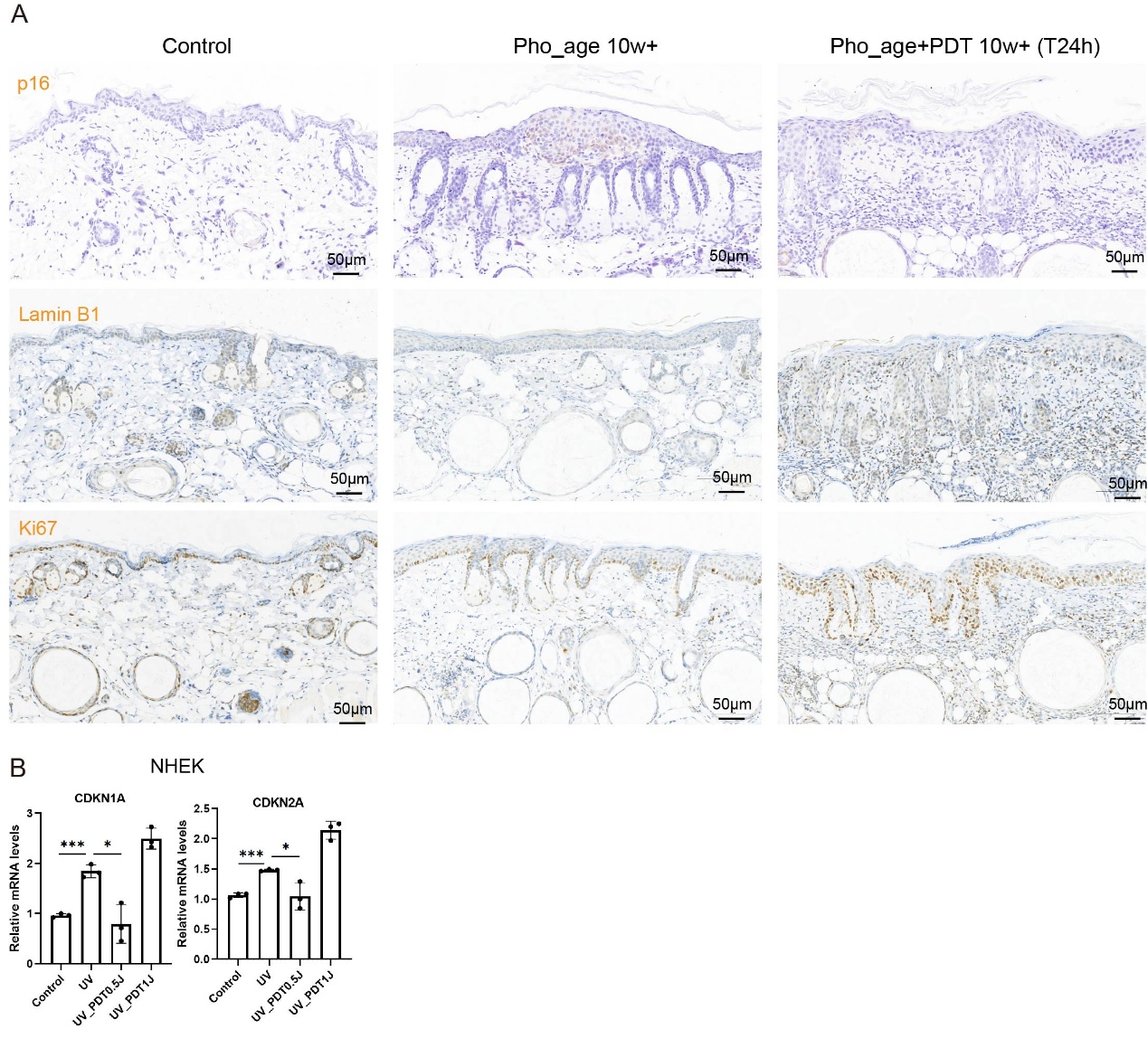
 **Supplementary figure 5.** A) Representative p16-stained, lamin B1-stained and ki67-stained sections of mice skin tissues. B) The mRNA levels of cellular senescence-related genes in normal human epidermal keratinocytes (NHEK). *P<0.05, **P<0.01, ***P<0.001.


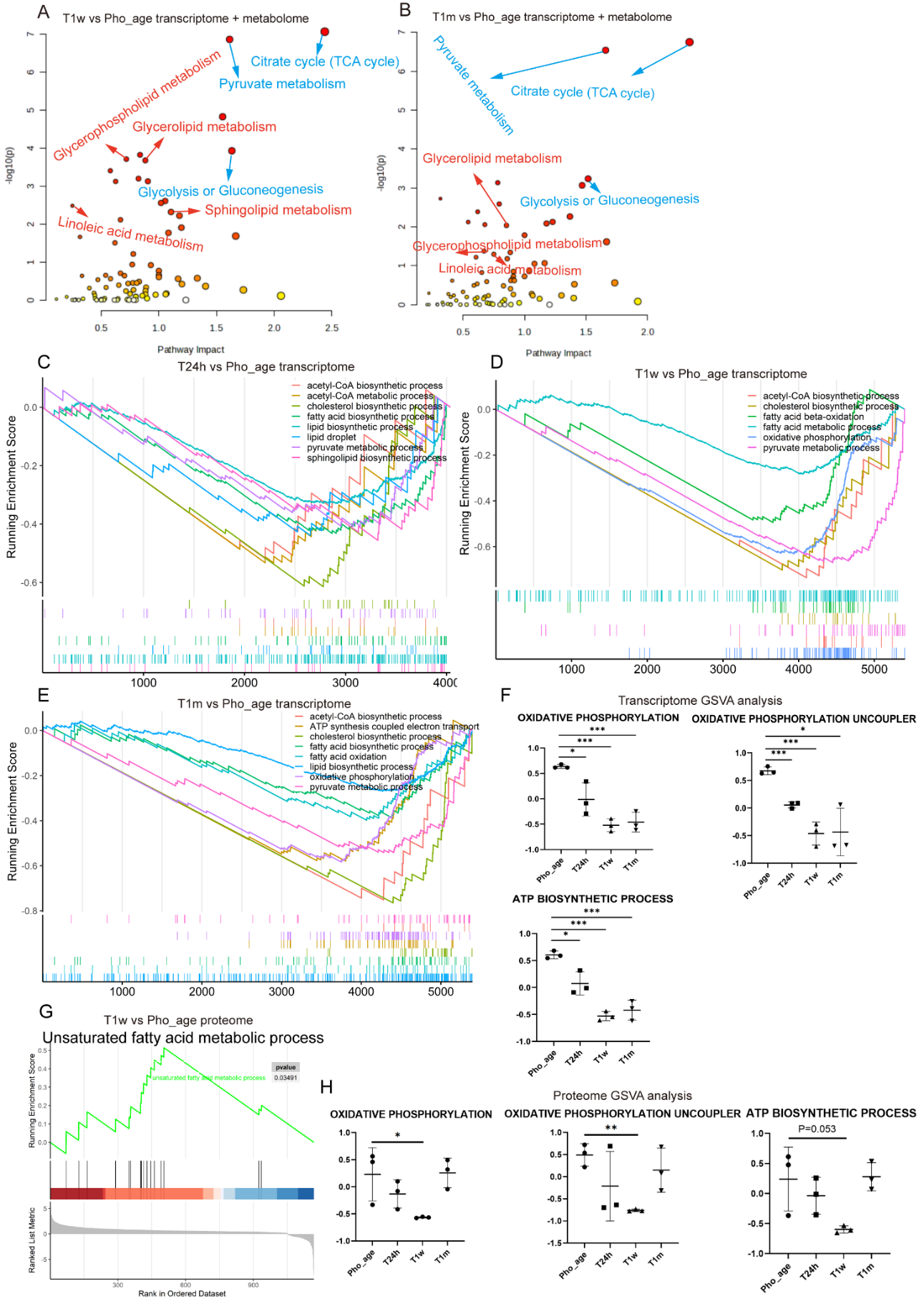


**Supplementary figure 6.** A, B) Combined analysis of transcriptome and metabolome performed by MetaboAnalyst. C-E) GSEA analysis of DEGs in different groups (P< 0.05). F) GSVA of OXPHOS-related pathways in transcriptomics. G) GSEA analysis of DEPs between T1w and Pho_age. H) GSVA of OXPHOS-related pathways in proteomics. *P<0.05, **P<0.01, ***P<0.001.

**
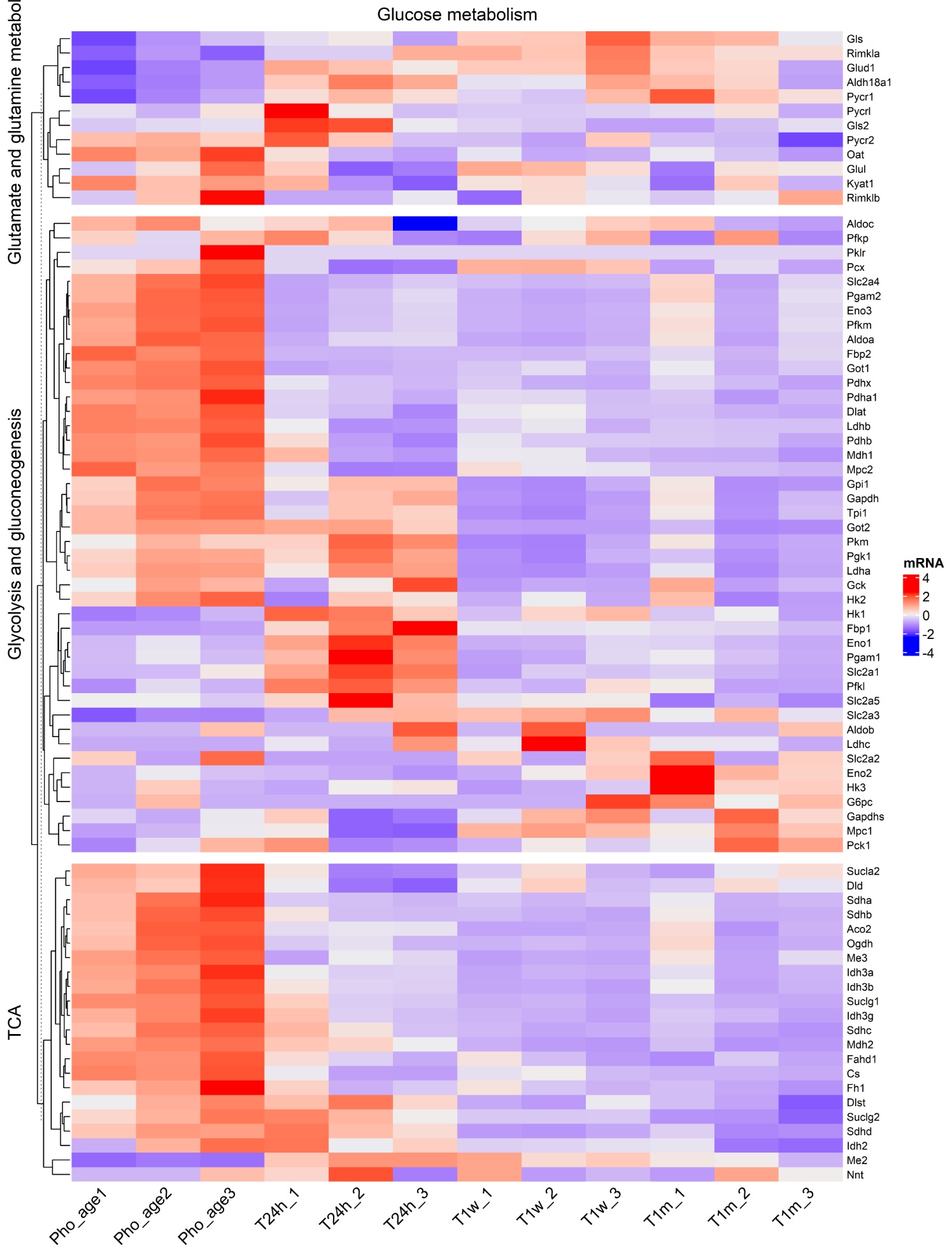
**

**Supplementary figure 7.** Heatmap of glucose metabolism related genes in transcriptomics.

**
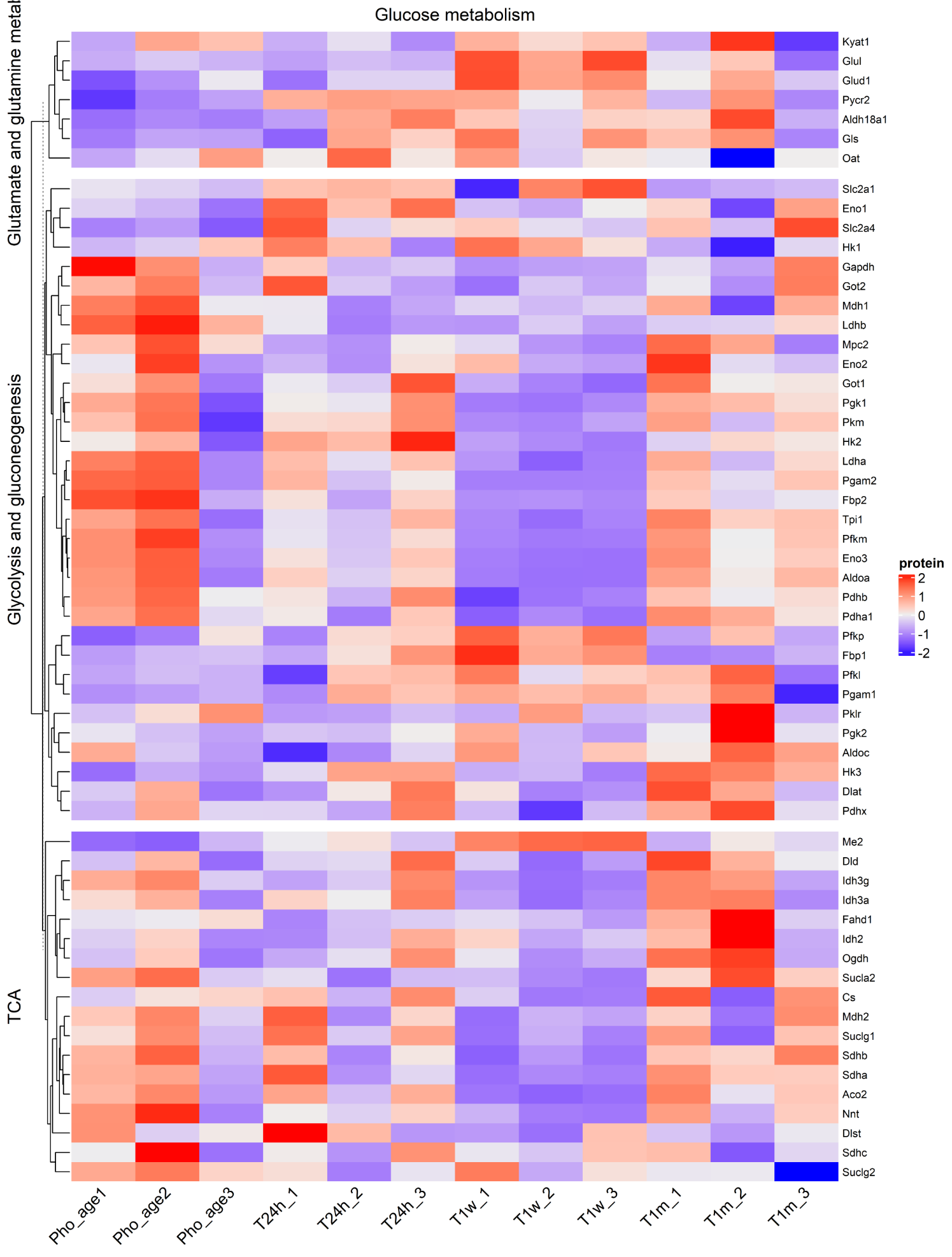
**

**Supplementary figure 8.** Heatmap of glucose metabolism related genes in proteomics.


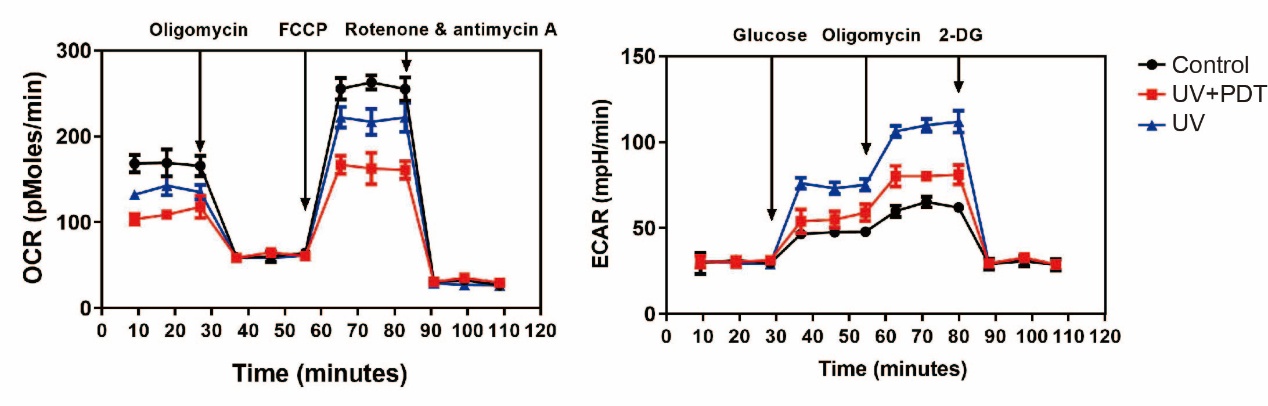


**Supplementary figure 9.** Seahorse assay-derived line graph illustrating oxygen consumption rate (OCR) and extracellular acidification rate (ECAR) dynamics in HDF cells. **
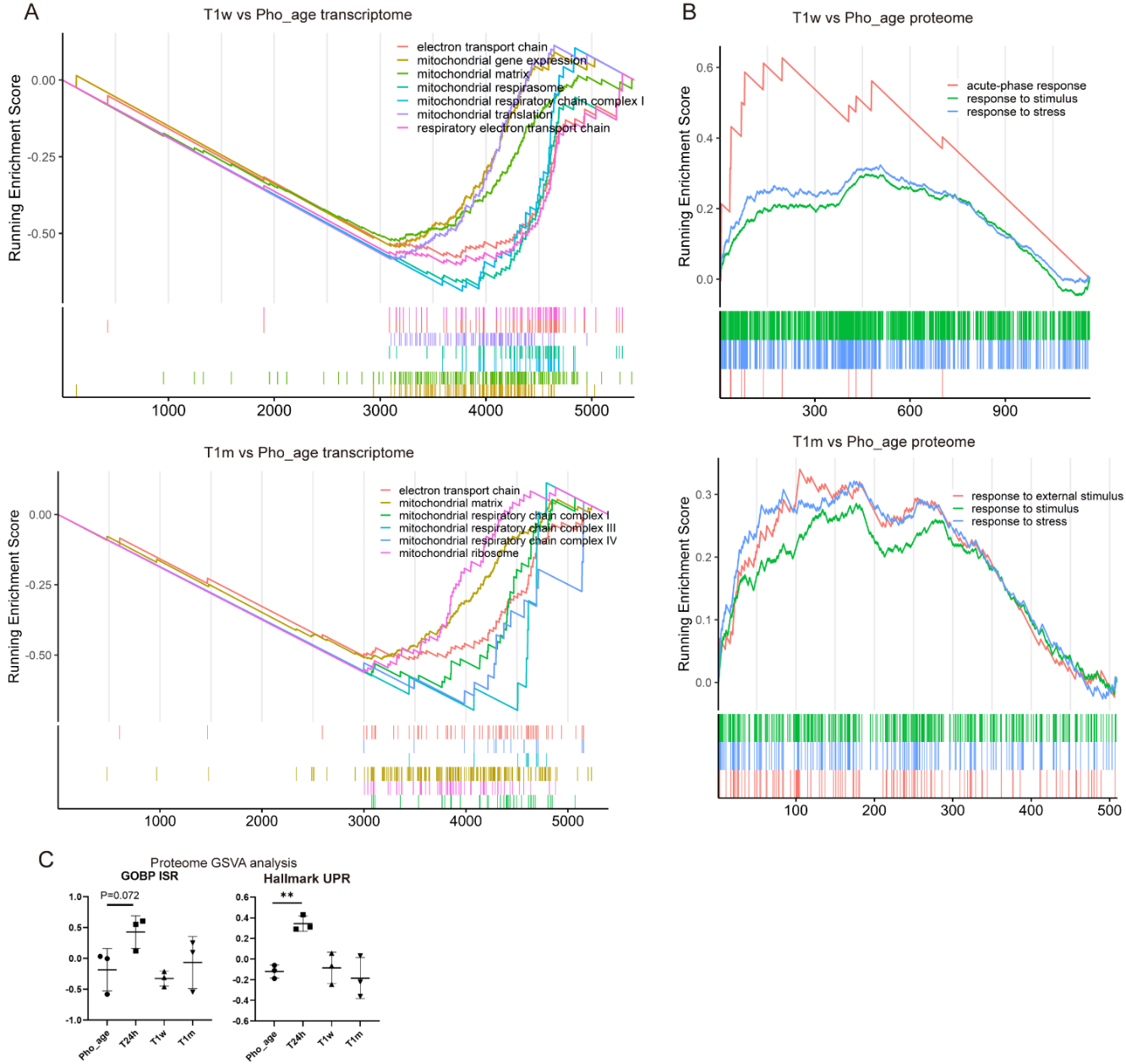
**

**Supplementary figure 10.** A, B) GSEA analysis of DEGs and DEPs in different groups (P< 0.05). C) GSVA of GO and hallmark pathways in proteomics. **P<0.01.

**
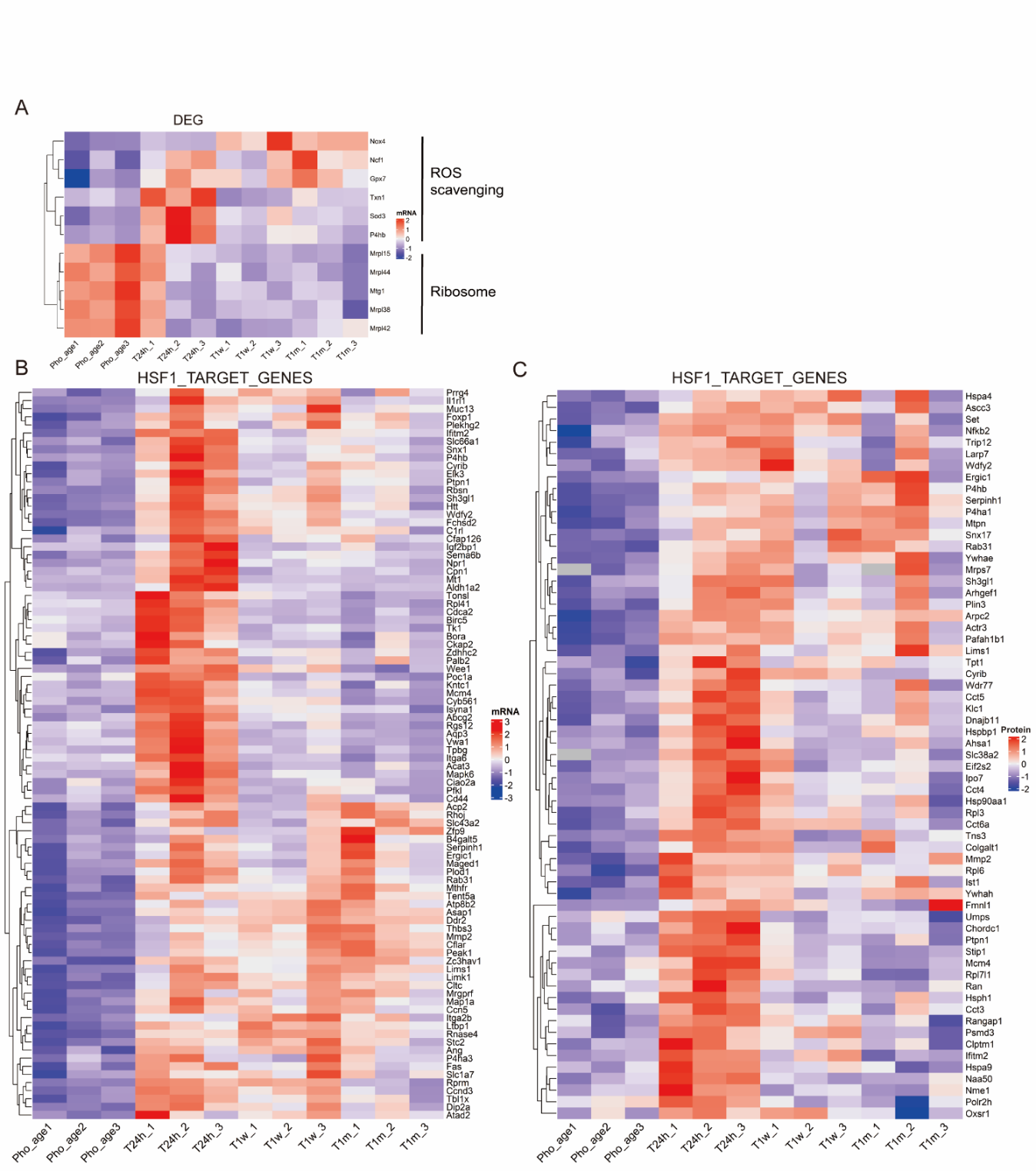
**

**Supplementary figure 11.** A) Heatmap of DEGs in different groups in transcriptomics. B, C) Heatmaps of DEGs and DEPs of HSF1 target genes in different groups.
